# High-Energy Batteries: Beyond Lithium-Ion and Their Long Road to Commercialisation

**DOI:** 10.1007/s40820-022-00844-2

**Published:** 2022-04-06

**Authors:** Yulin Gao, Zhenghui Pan, Jianguo Sun, Zhaolin Liu, John Wang

**Affiliations:** 1 Department of Materials Science and Engineering, National University of Singapore, Singapore, 117574 Singapore; 2 ST Engineering Advanced Material Engineering Pte. Ltd., Singapore, 619523 Singapore; 3 Institute of Materials Research and Engineering, Agency for Science, Technology and Research (A*STAR), Singapore, 138634 Singapore; 4 School of Materials Science and Engineering, Tongji University, Shanghai, 201804 China

**Keywords:** High energy density, Beyond lithium-ion batteries, Multivalent-ion batteries, Conversion electrode materials, Electrolyte

## Abstract

Rechargeable batteries of high energy density and overall performance are becoming a critically important technology in the rapidly changing society of the twenty-first century. While lithium-ion batteries have so far been the dominant choice, numerous emerging applications call for higher capacity, better safety and lower costs while maintaining sufficient cyclability. The design space for potentially better alternatives is extremely large, with numerous new chemistries and architectures being simultaneously explored. These include other insertion ions (e.g. sodium and numerous multivalent ions), conversion electrode materials (e.g. silicon, metallic anodes, halides and chalcogens) and aqueous and solid electrolytes. However, each of these potential “beyond lithium-ion” alternatives faces numerous challenges that often lead to very poor cyclability, especially at the commercial cell level, while lithium-ion batteries continue to improve in performance and decrease in cost. This review examines fundamental principles to rationalise these numerous developments, and in each case, a brief overview is given on the advantages, advances, remaining challenges preventing cell-level implementation and the state-of-the-art of the solutions to these challenges. Finally, research and development results obtained in academia are compared to emerging commercial examples, as a commentary on the current and near-future viability of these “beyond lithium-ion” alternatives.

## Highlights

- Fundamental rationalisation for high-energy batteries.
- Newly emerging and the state-of-the-art high-energy batteries vs. incumbent lithium-ion batteries: performance, cost and safety.
- Closing the gap between academic research and commercialisation of emerging high-energy batteries, and examination of the remaining challenges.

## Graphical abstract

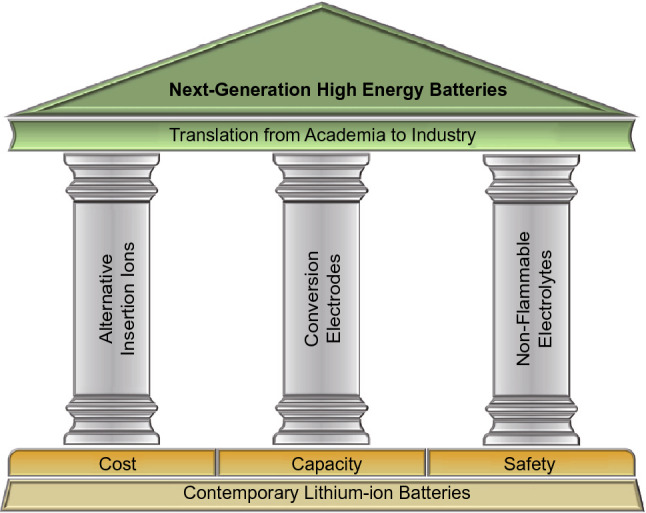

## Introduction

### Background

The battery, famously invented by Alessandro Volta in 1800 [1], is an electrochemical device that converts chemical energy to electrical energy. Redox reactants are stored in the electrodes, separated by an electronically insulating but ionically conducting electrolyte, with their reaction driving electrons through an external circuit during discharge. In a rechargeable system, simply applying a sufficient potential to the electrodes reverses the reaction and converts electrical energy to chemical energy. Rechargeable batteries have changed substantially in architecture, chemistry and performance since the initial invention. Regardless of these changes, the ability to reversibly store and release electrical energy on demand in a mobile package has had an undeniably significant impact on society, with off-the-grid electrical devices being ubiquitous all around the world. The role that they play is becoming even more important, as the depletion of fossil fuels and rapid climate change urgently call for clean, renewable sources of energy that will need to be stored in various electrical energy storage devices, including batteries, and especially so for mobile applications.

**Fig. 1:**
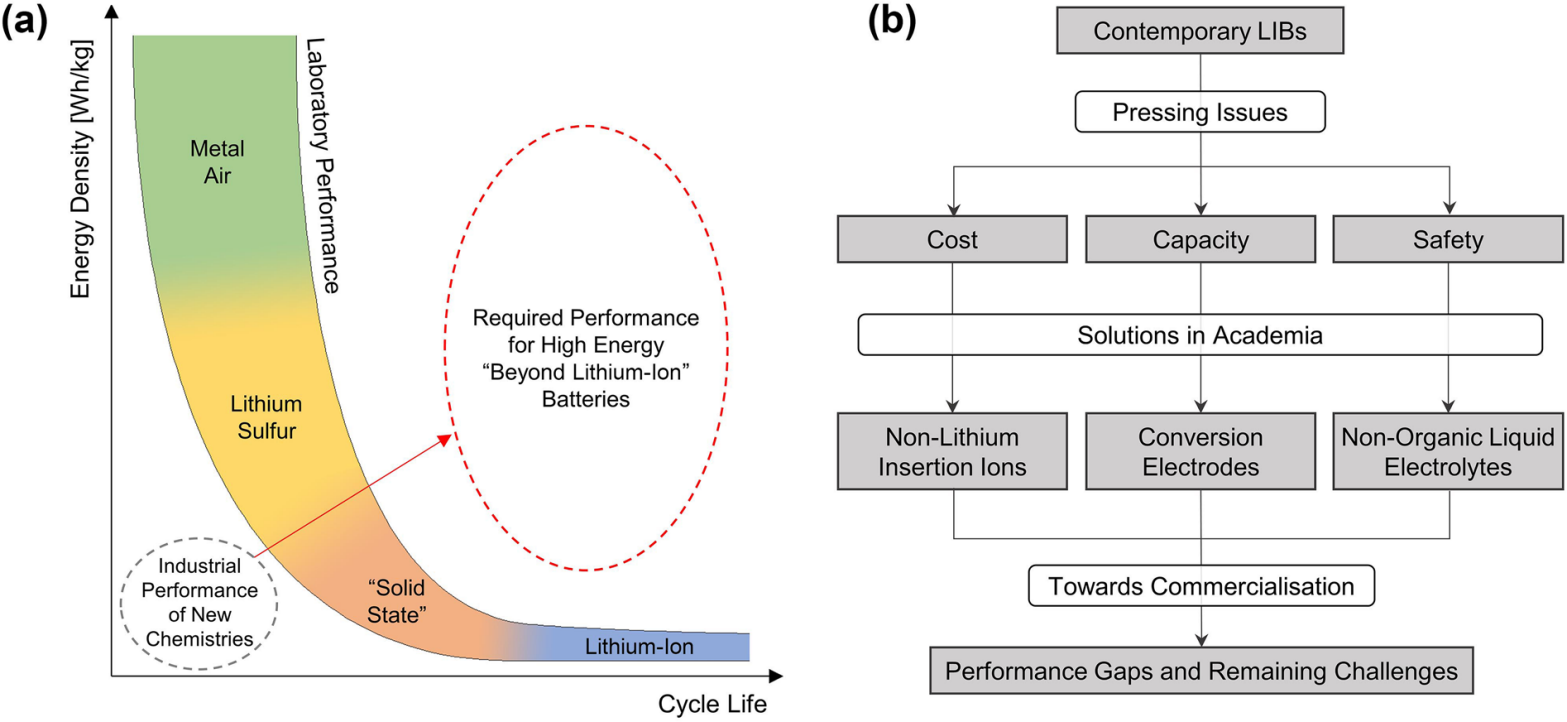
**a** General performance for LIBs and popular new chemistries along with emerging commercial examples of the latter, compared with the region of performance required by future applications. **b** Flowchart describing the sequence of content for this review

While other factors such as power capacity, cyclability, price and operating temperature are important, the perennial problem that batteries face is insufficient energy density,^1^ where battery designers are often engaged in an unwitting arms race with device designers that introduce ever more powerful devices to take advantage of ever more energy-dense batteries. Over the past few decades, lithium-ion batteries (LIBs) have emerged as the dominant high-energy chemistry due to their uniquely high energy density while maintaining high power and cyclability at acceptable prices. However, issues with cost and safety remain, and their energy densities are becoming insufficient with the rapid trend towards electrification of the transport and energy industries. There is thus an increasingly urgent need for better LIBs and “beyond lithium-ion” alternatives that are safer, cheaper and higher capacity while maintaining sufficient longevity and power capacity to address new application demands. A large variety of potential candidates have emerged as a result, including sodium and multivalent ion, lithium–sulphur, metal–air and solid-state batteries among others. While many of them have demonstrated potentially higher capacities or lower costs, this often comes at a large expense in cyclability. A large gap also remains between academia and industry, and emerging commercially available examples of these new alternatives are generally falling short of their laboratory counterparts in all performance aspects (Fig. 1a). As a result, despite encouraging academic progress, LIBs currently still account for nearly the entire high-energy battery market [2].

Nonetheless, recent progress in the field has been undeniably rapid, with an average of nearly 30,000 papers published globally per year for the past decade, covering a wide variety of different chemistries, architectures and applications. However, while numerous excellent reviews have been published on individual aspects of the field (e.g. on different battery types), holistic considerations of the entire field in the context of high-energy “beyond lithium-ion” batteries, including industrial developments, are absent. This review thus aims to rationalise and deconvolute these developments by returning to fundamental principles and examining the material characteristics that make a good high-energy battery, through which notable candidates are identified and correlated with trends in both academia and industry. A brief overview is then given regarding the main challenges of each system, strategies that are being adopted to tackle them and the extent of positive impact that these strategies have had on battery performance. Finally, cell performance in academia is compared to emerging industrial examples, where the differences are illustrative of the remaining barriers to successful commercialisation (Fig. 1b).

### Material Choices: Returning to Elements

Fundamental design of a high-energy battery begins with electrode material selection. In general, there are two types of electrode materials for batteries: insertion and conversion. Redox reactions occur in insertion electrodes via the insertion or removal of an active ion into or from an inactive host framework, with no change in the structure of the host beyond the inclusion or removal of the active ions and an associated volume change. This is also referred to as intercalation when it occurs in layered materials, and active ions are stored between layers. In contrast, conversion electrodes involve bond breakage and significant structural changes of the electrode materials during redox reactions. While distinctions are often made between the different types of bond-breaking reactions (e.g. dissolution, displacement, alloying and true-conversion reactions), these will broadly be referred to as conversion reactions for this review, as the challenges they face for use as batteries are largely similar.

**Fig. 2:**
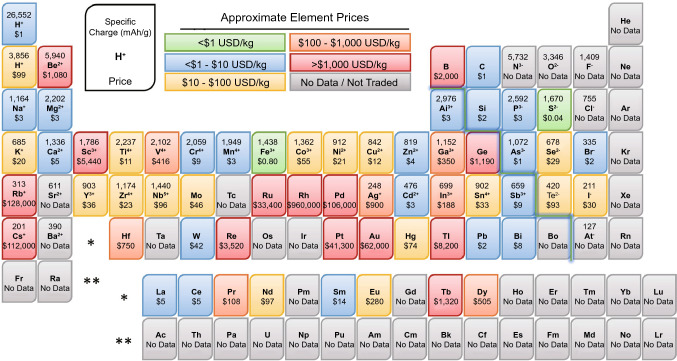
Periodic table of elements with price and charge capacity of each element at the indicated oxidation state. Price data retrieved from Shanghai Metals Market [3] (June 2021 spot price), Preismonitor [4] USGS Mineral Commodity Summaries [5], Institute of Rare Earths and Strategic Metals [6] and others [7, 8]*.* Gas prices vary substantially with location and storage method and are thus excluded

The energy density of an electrode is directly correlated with the charge capacity^2^ and redox potential of the active species involved. Thus, the suitability of an element for use as a battery electrode material can be, to some extent, assessed with the periodic table (Fig. 2). Within the same group, elements positioned higher on the periodic table possess higher charge capacities as their ion charges are the same, but atomic mass decreases. Within the same period, ion charges increase more than proportionately to increases in atomic mass, likewise leading to higher charge capacity, although this trend is slightly tenuous at high atomic numbers as electronic structures become more complex. Changes in redox potential within a period are less systematic and are also dependent on the counter-electrode, but a general trend of increasing theoretical energy density towards the top and centre of the periodic table can nonetheless be observed. It is also clear that elements, rather than their compounds, should be used as the active species whenever possible, as compounds necessarily have higher molecular weights than elements, but usually without the benefit of higher charge or redox potentials.

Other factors are also important for industrially relevant battery chemistries. First, the element should preferably be non-gaseous at operational temperatures for ease of handling and cell design. While gaseous batteries do exist in the form of fuel cells, the need to store the reacting gases in high-pressure vessels can substantially decrease the energy density of the energy storage system as a whole. The element should also be not overwhelmingly expensive or toxic to humans or the environment. It should be noted that the actual fabrication cost of an electrode can differ substantially from the element prices shown in Fig. 2, as the electrodes are usually not synthesised from the pure element, and element prices can also vary significantly with time, quantity and purity. Nonetheless, Fig. 2 offers an order-of-magnitude price gauge and a comparison of relative costs between the elements and their compounds.

By examining the periodic table with these factors in mind, one can have some ideas on good candidate materials for battery electrodes. Among the metallic cationic species, which are commonly used as both insertion ions and conversion anodes, lithium stands out as the only suitable element in Period 2. Beryllium, despite its high theoretical energy density, is unsuitable due to its high cost and toxicity. In Period 3, sodium, magnesium and aluminium are notable, and while their theoretical energy density is lower than lithium, they are considerably cheaper and thus have received substantial research interest [9–11]. A much larger number of cationic elements is available from Period 4. However, the high atomic masses of these elements results in lower theoretical energy densities than their Period 3 counterparts without significant cost benefits. The transition metal elements, despite their high charge capacities, also generally suffer from low redox potentials. Nonetheless, they do see use, but mostly in systems that are gradually facing obsolescence, such as the various nickel- and zinc-based batteries, or as insertion compounds for higher energy ions. They are also important for redox flow batteries [12], which will not be covered presently as energy density is usually not their primary goal.

Anionic species are rarely used as insertion ions due to them having higher atomic masses than cationic species of the same charge, as well as being generally more difficult to handle owing to their tendency to be gaseous at room temperature. Nonetheless, fluorine has been explored as a potential insertion ion [13]. For use as conversion cathodes, the monovalent halogen ions are gaseous and toxic, unlike their cationic analogue, the alkali metals. As a result, the light halogens like fluoride and chlorine are used as more benign, solid compounds instead, such as transition metal halides. However, the inclusion of inactive ions substantially reduces the theoretical charge capacity, resulting in halogens having substantially lower energy densities than those of chalcogens of the same period despite possessing higher redox potentials [14].

The chalcogens are generally more popular than the halogens. Oxygen can be used as a gaseous conversion cathode without storage issues, due to its unique combination of abundance in the atmosphere and high reactivity, allowing it to be harvested directly from ambient air [15]. Oxygen compounds are also commonly used as insertion electrodes due to their high stability [16]. Sulphur is the highest energy solid cathode material and thus also has received substantial research interest [17]. The pnictogens, despite having higher charge capacities than their chalcogen counterparts of the same period, are generally too stable to be used as conversion cathodes and are generally used as compounds in the form of insertion frameworks or conversion anodes instead [18, 19].

## Lithium-Ion Batteries: The Current Workhorse 

The dominance of LIBs for high-energy applications can in part be explained by lithium’s position in the periodic table, which gives it the highest charge capacity among suitable elements as previously shown, second only to hydrogen and beryllium. This, combined with the most negative standard reduction potential among all cationic elements of − 3.04 V, gives lithium an extremely high theoretical energy density, making it the obvious choice for a high-energy anode material. While the first rechargeable lithium batteries used lithium metal anodes with transition metal sulphide insertion cathodes, apparent safety issues prevented the commercial use of lithium metal anodes, and sulphide cathodes were gradually superseded by higher energy oxide cathodes [20]. The electrolyte is usually a lithium salt, such as LiPF_6_, dissolved in an organic solvent, the latter being necessary due to the typical operating potentials of lithium batteries far exceeding the electrochemical stability window of water.

### Anodes

Commercial LIBs nearly invariably employ a graphite intercalation anode, with lithium ions being stored between individual graphite layers. The fully lithiated state is of the composition LiC_6_, leading to a theoretical charge capacity of 339 mAh g^−1^ including lithium mass (372 mAh g^–1^ excluding lithium mass). Graphite is suitable for this application due to both sufficiently high electronic and lithium ionic conductivities that lead to low redox overpotentials. In addition, the volume expansion upon lithiation is fairly modest at around 10% [21], limiting electrode damage and allowing cycle lives of up to several thousand. Finally, its potential against metallic lithium is only 0.2 V [22], allowing for high energy densities at the cell level due to high cell voltages. However, this small potential difference also limits the charge rate, as lithium metal plating and associated dendritic growth can easily occur [23].

Lithium titanate (LTO), in the form of Li_4_Ti_5_O_12_, is another insertion anode material that has been commercialised. The fully lithiated state is of the composition Li_7_Ti_5_O_12_ [24], leading to a theoretical charge capacity of 167 mAh g^−1^ including lithium mass (175 mAh g^−1^ excluding lithium mass), which is substantially lower than carbon. LTO has a potential of approximately 1.55 V against metallic lithium, allowing for higher charge/discharge rates compared to graphite anodes due to the avoidance of lithium metal plating. It also has a lower tendency to form insulating solid electrolyte interphases, allowing the usage of higher surface area electrodes that further increase current capabilities. In addition, it produces nearly no volume change upon lithiation, improving cyclability [25]. However, the higher cost of LTO, as well as the higher reduction potential and lower theoretical charge capacity, which together lead to a significantly lower energy density, results in LTO usually being reserved for applications that require rapid charging or very long cycle lives.

### Cathodes 

A fairly large number of different insertion cathodes have been explored for LIBs, most of which are transition metal chalcogenides. However, over the past few decades, three main types have emerged as the frontrunners, all of which are oxygen-containing transition metal compounds. In general, insertion and removal of the lithium ion is balanced by reduction and oxidation of the transition metal ion, meaning the remaining ions in the compound are inactive and present only as structural stabilisers.

**Fig. 3:**
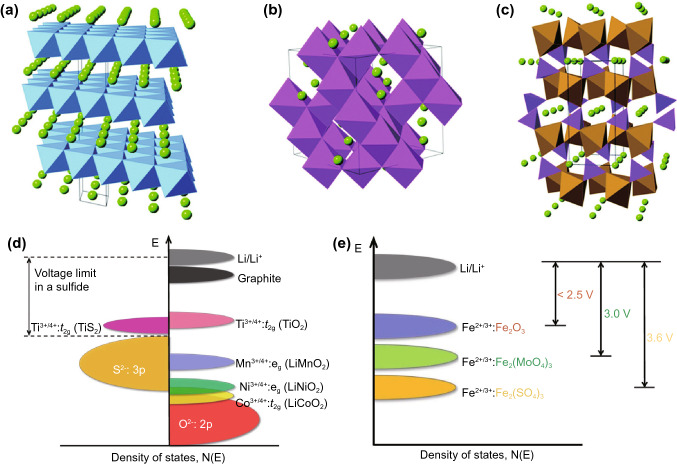
Schematics of the common LIB cathodes, **a** layered LiCoO_2_, **b** spinel LiMn_2_O_4_ and **c** olivine LiFePO_4_, with green atoms representing lithium [34]*.* Creative Commons License (CC BY 3.0). **d** Relative positions of the energy bands of different elements in LIB, showing the higher stability of Mn and Ni with oxygen compared to Co. **e** Illustration of the inductive effect of polyanions in increasing the cell voltage relative to lithium metal [16]. Creative Commons License (CC BY 4.0)

The first among these are layered transition metal oxides of the formula LiMO_2_, where the structure is idealised as close-packed planes of oxygen atoms between alternating layers of lithium and the transition metal. LiCoO_2_ (LCO, Fig. 3a) was the first to see widespread commercial use; however, its energy capacity is limited by the tendency for oxygen release above approximately 50% lithiation, due to the substantial overlap between the Co^3+^/Co^4+^ and O^2-^ densities of states. This means that only ~140 mAh g^−1^ out of the 273 mAh g^−1^ theoretical capacity can be accessed [16].

Other transition metal ions such nickel and manganese do not face the same issue to such an extent (Fig. 3d). However, LiMnO_2_ suffers from poor structural stability in the form of a layered-to-spinel transition during the charge/discharge process [26], and while LiNiO_2_ is stable during operation, it is unstable during the synthesis process into a well-ordered layered oxide [27]. Hence, state-of-the-art layered oxide cathodes are of the form LiNi_*x*_Co_*y*_Mn_*z*_O_2_ (NCM), where *x* + *y* + *z* = 1 and the three transition metal ions stabilise each other through *d*-band interactions. NCM cathodes are typically 60–80% nickel, with approximately equal remaining proportions of cobalt and manganese, and the general trend is to continue increasing the nickel content to improve energy capacity as well as minimise the use of expensive cobalt [16]. Replacing Mn with Al, resulting in LiNi_*x*_Co_*y*_Al_z_O_2_ (NCA) cathodes, can also achieve a similar stabilisation effect [28]. NCA cathodes are generally more resistant to phase transformations and dissolution than NCM, but more susceptible to mechanical pulverisation [29].

The second type of oxide cathodes are spinel oxides, for which LiMn_2_O_4_ (LMO, Fig. 3b) is the exemplary composition. Lithium-ion mobility in LMO is high, due to the presence of open channels for ion diffusion in all three dimensions, allowing for good current capabilities but not significantly superior to layered oxides [30]. However, the higher inactive mass compared to layered oxides gives rise to a lower charge capacity, and the tendency for the cathode to dissolve in the electrolyte due to acid disproportionation of Mn^3+^ [31] leads to limited cycle life. As a result, the main advantage of LMO over NCM is a slightly lower cost arising from the avoidance of cobalt, and does not see as much commercial use.

The final type of oxide cathodes are the polyanion oxides, which were first investigated due to an inductive effect resulting in higher redox potentials compared to simple oxides (Fig. 3e) [16]. While there are a large number of possible compositions, the emergent one in industry has been olivine LiFePO_4_ (LFP, Fig. 3c). However, LFP actually shows a lower redox potential than LCO, NCM and LMO due to the usage of iron. In addition, it has both low electronic and lithium ionic conductivity, resulting in the necessity for practical cathodes to be manufactured as small particles and contain a greater proportion of inactive conductive carbon [25]. These factors lead to LFP having the lowest practical energy densities among the three common cathode types. Nonetheless, LFP has higher thermal stability than layered and spinel oxides, leading to higher thermal runaway temperatures, and thus is prioritised for use in safety-critical applications [32]. They can also demonstrate higher cycle lives than layered oxide LIBs [33], making them popular for grid energy storage.

### Challenges Faced by LIBs

**Fig. 4:**
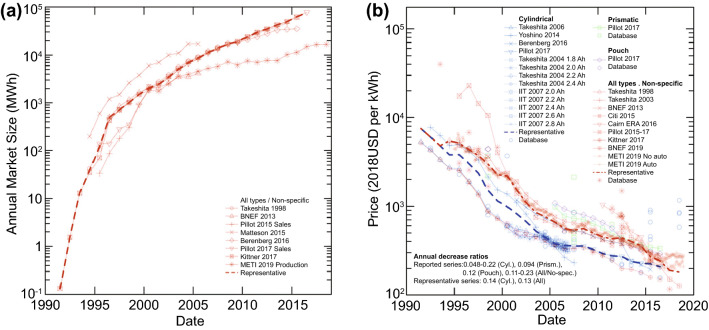
The change in **a** annual market size, and **b** price of LIBs since their introduction in 1991 [40]*.* Creative Commons License (CC BY-NC 3.0)

Commercial LIBs based on the aforementioned chemistries are capable of remarkably good performance compared to their nickel- and lead-based predecessors. NCM/Graphite cells can discharge at rates of up to 10C with energy densities of around 200 Wh kg^−1^ [35]. Under deep, slow discharge, energy densities can be as high as 280 Wh kg^−1^ [36]. Cycle life is also excellent, reaching as high as 6000 with 80% capacity retention [37] if cycled within a limited voltage window. LTO cells are capable of even higher cycle lives that can exceed 10,000 [38, 39]. These characteristics have propelled LIBs to significant popularity over the past three decades, with the market size in terms of total energy increasing by close to 6 orders of magnitude since their first introduction in 1991 **(**Fig. 4a**)** [40]. As of 2019, nearly the entire market for high-energy batteries is dominated by LIBs [2], with this rise apparently continuing as governments around the world increasingly encourage the adoption of electric vehicles and clean energy.

Nonetheless, a few issues remain to be addressed for these workhorse LIBs. Firstly, despite steadily decreasing in cost by 97% since 1991 **(**Fig. 4b**)** [40], LIBs remain fairly expensive as compared to the $80/kWh target set by the US Department of Energy’s Energy Storage Grand Challenge [41]. Additionally, as of 2021, the consumption of lithium by battery applications has already exceeded 70% of all global lithium consumption [5], and constantly rising demand has raised concerns on rising costs in the future due to potential resource depletion [42]. In addition to lithium, cobalt, frequently used in LIB cathodes, also faces a similar issue, with more than half of all global cobalt consumption being attributed to batteries [43].

Secondly, energy capacity also remains insufficient. Despite the relatively high energy densities already achieved by LIBs, which have played a part in enabling many applications including powerful mobile devices and a new wave of consumer electric vehicles, they remain substantially inferior to internal combustion engines and fuel cells. This is true even for small vehicles where the weight of the engines and fuel tanks would be more significant [44], making their usage in power-hungry or weight-critical applications such as heavy vehicles or aircraft highly challenging.

Finally, safety is also a critical issue for both mobile and stationary storage applications. LIBs notably contain all necessary components of the fire triangle once thermal runaway temperatures are reached. Rapid cathode decomposition releases heat and oxygen, while the flammable organic electrolyte and graphitic anode act as fuel [45, 46]. Further, the initial source of heat is often a short circuit, which will continue to act as a heat source until the battery is fully discharged. These factors lead to LIB fires being extremely difficult to extinguish. While external cooling and fire suppression mechanisms can be adopted, these will add further cost and weight to the energy storage system as a whole.

## Addressing Cost: Beyond Lithium Active Ions

**Fig. 5:**
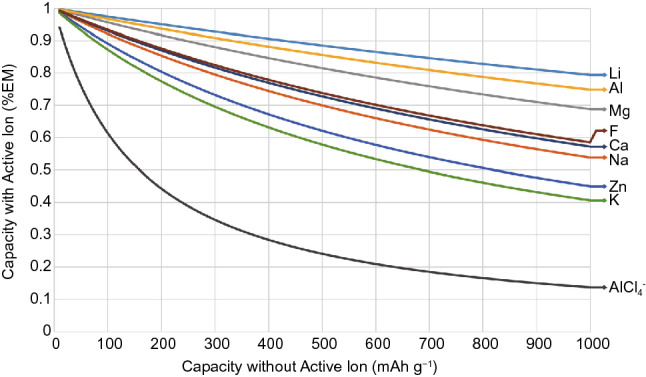
Theoretical charge capacity of an electrode calculated with both ion and electrode mass, plotted as a function of the charge capacity calculated with just the electrode mass (CEM), showing the detrimental effect of high ion mass on the charge capacity of an insertion electrode

An obvious solution to the issue of lithium cost and resource depletion is to use an alternative insertion ion. Although the mass of the active ion is only a small portion of the total mass of insertion electrodes, ion energy density is nonetheless important, especially for high-capacity electrodes (Fig. 5). Of the many potentially suitable ions identified previously through the periodic table, magnesium and aluminium stand out due to their high theoretical energy densities, second only to lithium. Sodium and zinc are also popular alternative ions, the former due to its very similar chemistry to lithium, and the latter due to its less negative standard reduction potential allowing it to be used more easily with aqueous electrolytes.

Several other ions have also been explored, which have plenty of academic novelty, but which are disadvantaged compared to the aforementioned four ions for industrial relevance. Potassium [47] and calcium [48] are popular, but currently offer uncertain benefits over their Period 3 counterparts. Fluorine has received attention due to its high redox potential and relatively low atomic weight. However, the theoretical energy density of the fluoride ion remains inferior to lithium, magnesium and aluminium, and the system faces significant difficulties in finding suitable electrolytes [13].

### Sodium-Ion Batteries

**Fig. 6:**
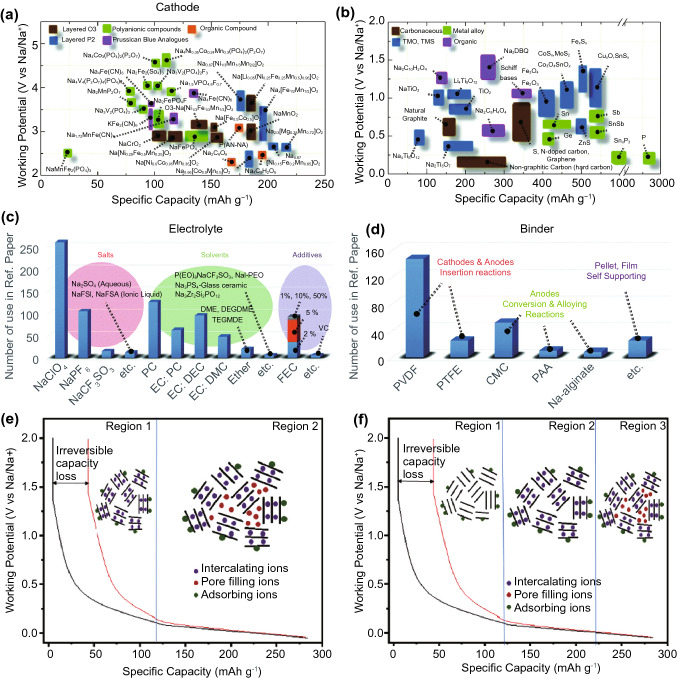
A non-exhaustive visual summary of selected **a** cathodes, **b** anodes, **c** electrolytes and **d** binders that have been explored for sodium-ion batteries [9]*.* Creative Commons License (CC BY 3.0). **e** Initial model for sodium ion insertion into hard carbon, attributing the sloping region to intercalation and low potential plateau to pore-filling. **f** A more recent model for sodium insertion into hard carbon, attributing the sloping region with defect binding, and the low potential plateau to intercalation and pore-filling [50]*.* Copyright 2020, Elsevier

The popularity of sodium as an insertion ion, aside from its low cost and high elemental abundance in the earth’s crust, also arises from it being an alkali metal like lithium, resulting in very similar chemical behaviour. In fact, sodium ion electrolytes (Fig. 6c), typically a sodium salt such as NaPF_6_ dissolved in an organic solvent, are nearly identical to lithium-ion electrolytes aside from the change from lithium to analogous sodium salts [49]. Anode and cathode compositions are also highly similar to those in LIBs, leading to rapid developments that have propelled sodium-ion batteries (SIBs) to near-commercial viability.

While oxide insertion anodes such as TiO_2_ exist (Fig. 6b), the dominant anodes for SIBs are carbon-based, like LIBs [50, 51]. However, unlike lithium, regular graphite is a very poor anode for sodium due to its larger ionic radius, resulting in negligible reversible capacity in typical organic solvents [52, 53]. Expanded graphite shows better results, which can be achieved either by direct synthesis for capacities as high as 284 mAh g^−1^ [54] or by co-intercalation of non-standard organic solvent molecules such as diglyme [55] for capacities in the region of 100–200 mAh g^−1^. Nonetheless, most of the explored carbon anodes for SIBs are based on non-graphitic carbon such as hard carbon. Sodium ion insertion proceeds first along a high potential, sloping region, followed by a low potential plateau. Initially, the sloping region was attributed to intercalation between graphene sheets in turbostratic domains in the hard carbon, while the low potential plateau was attributed to the filling of nano-pores located among these domains (Fig. 6e) [56]. However, more recent evidence points towards the sloping region being due to sodium ion binding with defect sites instead, with the low potential plateau being attributed to both turbostratic domain intercalation and nano-pore filling (Fig. 6f) [50]. Regardless of the mechanism, the more disordered structure of hard carbon compared to graphite allows it to achieve higher charge capacities, generally in the region of 200–300 mAh g^−1^. The charge capacity can be further increased to some extent by appropriate heteroatom doping, which increases the turbostratic interlayer spacing [57]. Soft carbons, with mixed graphitic and disordered domains, are able to achieve similar charge capacities as hard carbon, but with substantially higher current capabilities due to higher electronic conductivity.

SIB cathodes are likewise very similar to LIBs, with the majority consisting of layered and polyanion oxides with similar insertion mechanisms (Fig. 6a). It is then perhaps no surprise that one of the more promising full-cell SIBs recently reported is based on the familiar layered NCM chemistry [58]. However, the larger ionic radii of sodium ions mean they cannot be stored in the same lattice sites in layered oxides as lithium, resulting in phase transformations during sodium insertion and extraction that lead to stepped charge/discharge curves [59]. Remarkably, the larger ionic radius and higher atomic mass does not appear to affect the charge storage capacity as much as in anodes, and layered sodium oxides are able to achieve charge capacities similar to layered lithium oxides at around 200 mAh g^−1^ [60–62].

Polyanion oxides show greater differences between sodium and lithium analogues. Most notably, NaFePO_4_ is unstable in the olivine structure that is so popular for LFP batteries. Although the olivine phase can be synthesised in laboratories, it remains susceptible to phase transformations [63, 64] during charge/discharge and sodium diffusion coefficients are substantially lower than lithium due to the larger ion size [65]. As a result, many other types of polyanion oxides have been explored, including phosphates, fluorophosphates, pyrophosphates and sulphates. Among these, NASICON-type compounds are notable due to their high sodium ionic conductivity. Nonetheless, polyanion oxide cathode capacities are typically in the region of 100 mAh g^−1^, and no dominant composition appears to have emerged yet [9, 60, 61].

Other than the oxides, Prussian Blue Analogues (PBAs) are also popular sodium insertion cathodes by virtue of their open structures. The usage of PBAs as cathodes is complicated by the large amount of vacancies and coordinated water that arise from their synthesis processes, detrimentally affecting electrochemical performance. Nonetheless, these synthesis processes have since been refined sufficiently to allow cathode capacities generally in the region of 150 mAh g^−1^ [66], with exceptional examples exceeding 200 mAh g^−1^ [67]. Nonetheless, these charge capacities, along with generally similar redox potentials, means energy density remains lower than those of layered oxides. However, PBAs do not suffer from phase transitions the during charge/discharge process, leading to the possibility of longer cycle lives [68].

Unfortunately, the strong chemical similarities between sodium and lithium that have driven the rapid recent developments of SIBs is also a major disadvantage. As the electrodes and electrolytes are so similar, the lower redox potential and charge capacity of sodium ions mean the energy density of SIBs are invariably lower than their LIB analogues [69]. Hence, the only significant advantage the former can currently claim over the latter is potentially lower cell costs arising from the use of a lower cost active ion. Further cost savings can be achieved with the use of cheaper aluminium as anode current collectors in SIBs, as opposed to copper in LIBs. This is due to a low-potential reaction that occurs for aluminium with lithium, but not with sodium [42]. However, even this advantage is threatened by the constantly falling costs of LIBs.

### Aluminium-Ion Batteries

The attractiveness of aluminium-ion batteries (AIBs) stems from the very high charge capacity of the trivalent aluminium ion. At 2976 mAh g^−1^, it is second only to lithium among the relatively low-cost elements that are solid at room temperature. This, in combination with a decent standard reduction potential of – 1.66 V, results in a very high theoretical energy density. However, the trivalency also results in a very high surface charge density and thus high stability of compounds formed. This results in generally lower salt solubility and ion mobility, and more difficult insertion into and extraction from electrodes than monovalent and divalent ions. Notably, the low salt solubility means sufficient ionic conductivity generally cannot be achieved for the salt-in-solvent type organic electrolytes used in SIBs and LIBs [11].

Research and development of AIBs is far less mature than SIBs and LIBs, and most efforts have been focused on finding suitable electrolytes and characterisation of the insertion process in general. The latter is especially important in AIBs as the aluminium ion can insert into an electrode in three different valence states, with only the trivalent ion able to approach the theoretical charge capacity. Hence, while the potential difference against metallic aluminium of some insertion compounds such as TiO_2_ [70, 71] is small enough for them to be used as anodes, the vast majority of AIBs in literature simply use metallic aluminium as the anode.

**Fig. 7:**
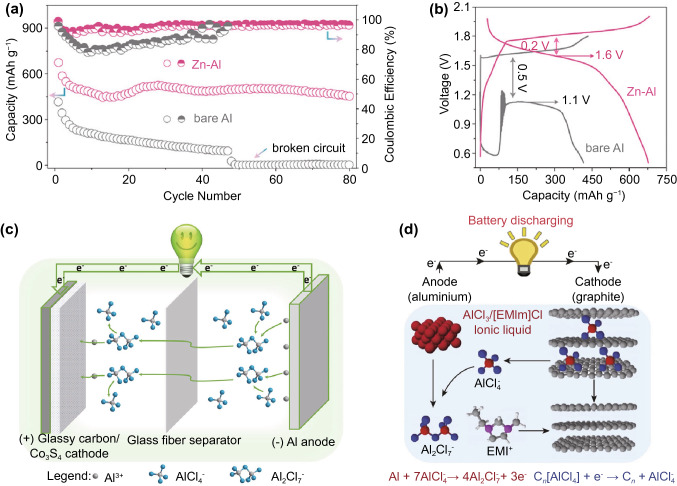
**a** Galvanostatic discharge curve of a high performance MnO_2_ cathode with Zn-Al anode, with the high energy density arising from both high charge capacity and **b** high discharge voltage [80]. Copyright 2020, American Chemical Society. **c** Schematic of the insertion of the high capacity Al^3+^ ion [81]*.* Copyright 2019, Elsevier. **d** As a comparison, schematic of the insertion of heavy AlCl_4_^−^ ion, commonly seen in carbon cathodes [82]*.* Copyright 2015, Springer Nature

With the difficulty of utilising organic electrolytes, an obvious substitute is aqueous electrolytes. However, aside from the usual issue of electrochemical breakdown of water, aqueous electrolytes also cause the formation of an impermeable layer of Al_2_O_3_ on the surface of the metallic aluminium anode, passivating it from the redox reactions necessary for battery operation. While this can be avoided with basic electrolytes, the aluminium anode corrodes in a basic environment instead, leading to continuous self-discharge [72]. As a result, the majority of AIBs use room temperature ionic liquid electrolytes, usually AlCl_3_ mixed with 1-ethyl-3-methylimidazolium chloride ([EMIm]Cl), which appears to facilitate the formation of an ionic-liquid-rich solid electrolyte interphase (SEI) that simultaneously erodes Al_2_O_3_ and prevents its re-formation. Interestingly, this interphase has been found to persist even if the aluminium anode is subsequently transferred into aqueous electrolytes, allowing their use [73]. It should be noted that aluminium does not dissolve into AlCl_3_-based electrolytes as the trivalent Al^3+^ ion, but as AlCl_4_^−^ and Al_2_Cl_7_^−^ [74]. However, the numerous reports of Al^3+^ cathodic insertion (Fig. 7c) in AlCl_3_ electrolytes [75–79] imply that these monovalent anions are subsequently converted back into Al^3+^ at the cathode in a process that appears to be neither very well studied nor understood at the moment.

Most AIB cathodes reported so far are based on a variety of different transition metal chalcogenides, including oxides, sulphides and selenides, although PBA cathodes are also known. A common issue that plagues the majority of these cathodes is a low redox potential against the aluminium anode, with average cell discharge voltages of 1 V or even less, severely limiting the energy density [11, 72, 74, 83]. This is an issue that will be further exacerbated if insertion anodes are used. The highest energy densities achieved so far, as high as 736 Wh kg^−1^ for cathode mass only with a zinc/aluminium alloy anode [80], are with MnO_2_ cathodes (Fig. 7a, b). However, while the generally accepted mechanism is aluminium ion insertion, recent evidence points towards a conversion reaction involving soluble Mn^2+^ instead [84]. Conversion reactions are generally less reversible, and could contribute towards the reported cycle lives of MnO_2_ cathodes not exceeding 100, echoing the challenges faced by LiMnO_2_ due to structural instabilities.

Overall, AIBs remain in the early stages of development. While ionic liquid electrolytes work in a laboratory environment, they are highly corrosive and expensive, and thus difficult to commercialise. A sufficiently stable, high voltage cathode also needs to be found before a commercially useful battery with acceptable energy density and cycle life can be realised.

While not strictly AIBs, chloroaluminate batteries based upon the insertion of AlCl_4_^-^ ions into usually carbon-based cathodes (Fig. 7d) have also seen rising popularity in recent years, taking advantage of the presence of these ions in AlCl_3_-[EMIm]Cl electrolytes. High cell voltages of around 2 V and respectable cathodic charge capacities of around 200 mAh g^−1^ have been reported [85, 86]. However, it must be noted that the charge capacities do not usually consider the mass of the heavy AlCl_4_^−^ ion, which has a theoretical charge capacity of only 159 mAh g^−1^. As such, the viability of these chloroaluminate ion chemistries as high-energy batteries remains unclear [87].

### Magnesium-Ion Batteries

Like aluminium, the attractiveness of magnesium-ion batteries (MIBs) arises from a high theoretical energy density. The lower charge capacity of 2202 mAh g^−1^ is compensated by a substantially more negative standard reduction potential of − 2.37 V, resulting in a theoretical energy density against the standard hydrogen electrode (SHE) that is actually slightly higher than aluminium. The MIB anode scene is somewhat more vibrant than for AIBs, with a large number of group 14 and 15 alloying anodes, primarily tin and bismuth, having been explored [88]. Nonetheless, at the cell level, most MIBs use magnesium metal anodes. This is in part due to magnesium metal showing a lower propensity for dendrite growth than other metals by virtue of its high self-diffusion rates. However, despite common claims on the contrary, magnesium metal is not in fact immune to dendritic growth, which can occur in unfavourable electrolytes or at high current densities [89].

**Fig. 8:**
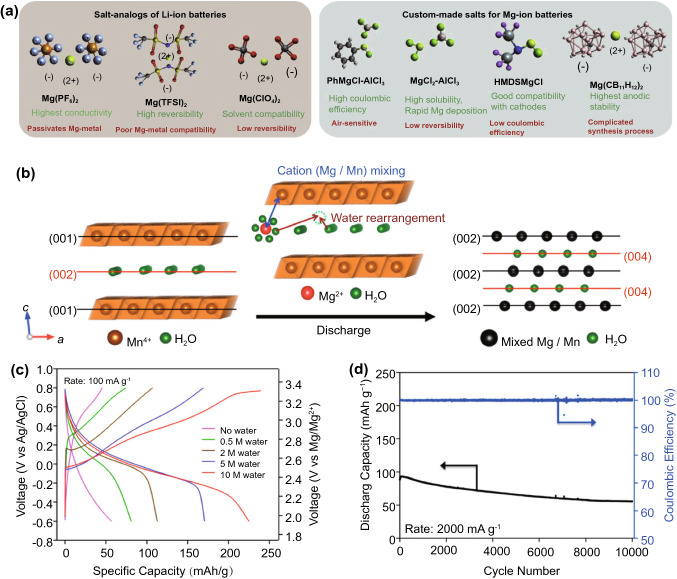
**a** Illustration and comparison of a selection of magnesium salts used in organic MIB electrolytes [92]*.* Copyright 2019, Elsevier. **b** Schematic of the co-insertion of water and Mg^2+^ ions into a birnessite MnO_2_ cathode, and electrochemical performance showing **c** improved capacity with higher water concentration and **d** excellent cycle stability of the cathode [94]*.* Copyright 2015, American Chemical Society

The lower surface charge of the Mg^2+^ ion means it does not face as large of a solubility issue as Al^3+^, and salt-in-solvent type organic electrolytes are viable. However, unlike LIBs, where reactions between the electrodes and organic electrolytes form an SEI that protects against further reaction but still allows lithium-ion conduction, the SEIs formed between MIB electrodes and simple magnesium salts in conventional organic solvents insulate against magnesium ion conduction due to the higher ionic charge density [90]. Instead, magnesium organohaloaluminate electrolytes are typically used, formed by the reaction of a Lewis acid, commonly an aluminium chloride, with an alkyl or aryl Grignard reagent in an organic solvent (Fig. 8a). Grignard reagents alone can also be used, but they show a relatively small electrochemical stability window, and reactions with the chloride Lewis acid improves stability. However, these electrolytes are often corrosive to typical battery housing materials, and halogen-free electrolytes are being explored to address this issue [91–93].

The first rechargeable MIBs were demonstrated with Chevrel phase molybdenum sulphide cathodes [95]; however, they suffer from both low charge capacities and low redox potentials against the magnesium anode. Since then, similar to AIBs, a variety of different transition metal chalcogenides and PBAs have been explored [10, 96–99]. While no dominant chemistry has yet emerged, vanadium [100] and manganese oxides [101] have received comparatively more attention than others. Energy densities of as high as 800 and 650 Wh kg^−1^ based on cathode mass only have been reported in layered V_2_O_5_ [102] and MnO_2_ [94]_,_ respectively, with high discharge voltages of around 3 V. Interestingly, in both cases, the high performance is attributed to the incorporation of crystal water into the layered structure, which shields the high charge of the Mg^2+^ ion that normally results in poor ionic mobility [96]. In the case of MnO_2_, the crystal water also appears to stabilise the cathode against the commonly faced dissolution issue, allowing a remarkably high cycle life of 60% capacity retention after 10,000 cycles, although at a high current that leads to substantially lower capacity (Fig. 8b–d). Unfortunately, most high-energy cathodes reported for MIBs were characterised with three-electrode cells, and it is unclear if the high potentials measured with Ag/Ag^+^ reference electrodes can be maintained in the two-electrode configuration of a conventional cell.

Overall, MIBs continue to suffer from generally the same issues as AIBs. Electrolytes suitable for laboratory cells are now fairly commonplace, but examples suitable for commercialisation remain elusive. Gaps remain with the electrodes as well, as a sufficiently stable and energy-dense combination has yet to be demonstrated at the cell level.

### Zinc-Ion Batteries

Zinc may at first appear to be a puzzling choice for an active ion due to its low energy density of 622 Wh kg^−1^ against the SHE, an order of magnitude less than lithium, aluminium and magnesium, due to both its low charge capacity and standard reduction potential. However, decent potentials can in fact be achieved at the cell level with appropriate cathode selection, and the low standard reduction potential makes it attractive for use with aqueous electrolytes, as far less stabilisation is required at the anode than the higher energy elements. Metallic zinc anodes also have a history as long as the entire field of batteries, dating back to Alessandro Volta’s invention of the voltaic pile, allowing researchers a large body of prior work from which to draw inspiration. Hence, while non-aqueous zinc-ion batteries (ZIBs) [103] and insertion anodes are known [104], ZIBs typically use metallic zinc anodes and aqueous electrolytes, consisting of various zinc salts dissolved in water, with most variations occurring at the cathode.

**Fig. 9:**
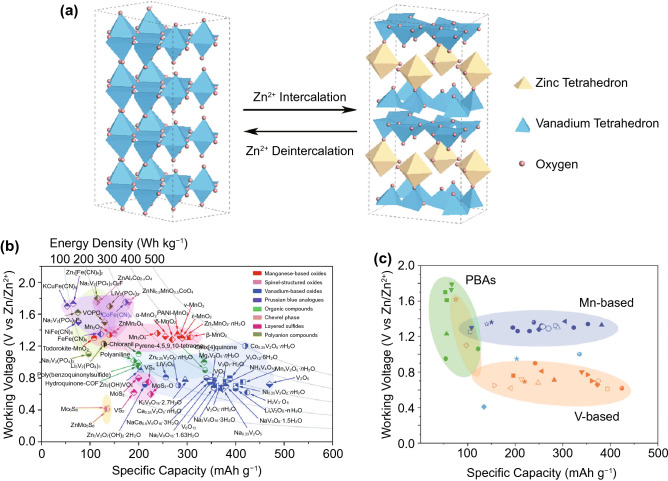
**a** Schematic of Zn ion insertion into a representative V_2_O_5_ cathode framework. **b, c** Cell voltages and charge capacity of various cathodes for ZIBs. Reproduced with permission [105, 107]*.* Copyright 2021, Elsevier. Copyright 2020, Royal Society of Chemistry

Popular ZIB cathodes can be classified into three familiar categories: manganese oxides, vanadium oxides and PBAs (Fig. 9b, c) [105–107]. The highest energy densities are again achieved with MnO_2_ of various structures, with charge capacities generally in the region of 200–400 mAh g^−1^ and operating voltages of around 1.3 V. However, like with aluminium ions, recent evidence points towards a dissolution conversion reaction rather than Zn^2+^ insertion [108–110]. The same structural instabilities and dissolution issues as with other active ions also remain present, leading to short cycle lives of less than 100 cycles in general. Nonetheless, a remarkably high cycle lives of more than 90% capacity retention after more than 1000 cycles have been achieved with hollandite-MnO_2_, enabled by reduced graphene oxide coatings [111], or the suppression of MnO_2_ dissolution via Mn-salt inclusion in the electrolyte [112]. However, there is some debate on whether the salt inclusion truly suppresses dissolution, or instead improves cyclability by providing buffer ions for the Mn^2+^ conversion reaction [84].

The charge capacities of vanadium oxide cathodes are likewise around the region of 200–400 mAh g^−1^, but energy density is limited by a generally low cell voltage of less than 1 V against metallic zinc. However, cyclability is generally superior to manganese oxides, with cycle lives frequently exceeding 1000 with more than 80% capacity retention, owing to the high reversibility of zinc insertion reactions (Fig. 9a). Similar to MIBs, incorporation of water into the vanadium oxide framework is often beneficial for performance [105, 107]. PBA cathodes have the highest operating voltages of up to 1.8 V [113] against metallic zinc, but charge capacity is low, at generally less than 100 mAh g^−1^ due to the same defect and coordinated water issues faced in SIBs.

Compared to MIBs and AIBs, ZIBs are closer to commercialisation, due to the more stable zinc anode allowing for the use of a wider variety of aqueous electrolytes along with the encouraging progress made with various cathodes. Nonetheless, the use of aqueous electrolytes limits the achievable voltages at the cell level, which will be discussed in greater detail in Sect. 5.1. The apparently attractive charge capacities achieved are also usually evaluated with cathode mass only, and inclusion of the mass of the heavy zinc ions can lower charge capacity by more than 30% (Fig. 5). This is far greater than the higher energy elements covered previously, and results in severely compromised energy densities of full cells.

### Other Metal-Ion Batteries

#### Potassium-Ion Batteries

Potassium-ion batteries (PIBs) have also been actively pursued as alternatives to LIBs and SIBs by virtue of their similar chemical behaviour. The potassium ion has a theoretical charge capacity of only 685 mAh g^−1^, substantially lower than that of sodium ion (1165 mAh g^−1^), putting it at a distinct disadvantage for high-energy applications. However, potassium does have two important redeeming features, the first being a significantly more negative standard reduction potential of − 2.93 V compared to the − 2.71 V of sodium, which may partially compensate for its lower charge capacity to high energy densities. Secondly, unlike sodium, potassium can intercalate into graphite to form KC_8_ [114]. Therefore, the anode can be made highly similar to those in commercial LIBs, which would be a merit for eventual commercialisation. Like SIBs, the dominant electrolytes in PIBs use potassium salts that are analogous to those used in LIBs, such as KF_6_, dissolved in an organic solvent [115], which is another boon for commercialisation.

Nonetheless, PIBs continue to face several challenges. For example, potassium ion insertion onto graphite is associated with a volume expansion of ~ 61% [116], substantially larger than lithium and which severely hinders cycle performance. Although some alternative anodes are available, such as hard and soft carbons, as well as non-carbonaceous materials, the cycle performance is generally in the hundreds rather than thousands achievable by LIBs and SIBs. The initial Coulombic efficiency also tends to be low, at less than 60%, due to the formation of undesirable SEIs that require electrolyte modification to resolve [115, 117, 118]. There are difficulties with the cathode as well. Although several SIB and LIB cathodes are useable in KIBs, such as PBAs and transition metal oxides, both the cyclability and charge capacity tend to be lower, due to the larger size and lower charge capacity of the potassium ion. In particular, the layered oxide cathode materials that have seen so much success in SIBs and LIBs can only achieve capacities in the region of 150 mAh g^−1^ when used in KIBs [119, 120], as compared to ~ 200 mAh g^−1^ in the former two. Although this can be partially compensated for by higher cell voltages than SIBs, they remain lower than those of LIBs. Finally, while cheaper than lithium, potassium is substantially more expensive than sodium. This means that the advantage of lower costs, which applies to SIBs over LIBs, is significantly diminished in the case of PIBs.

#### Calcium-Ion Batteries

Calcium is an alkaline earth metal, and like potassium, trades a slightly more negative standard reduction potential (− 2.84 vs. − 2.37 V) for a substantially lower ion charge density (1336 vs 2202 mAh g^−1^) as compared to its Period 2 counterpart, magnesium. This results in a significantly lower theoretical energy density against the SHE.

Calcium-ion batteries (CIBs) share many similar challenges with the other non-aqueous multivalent batteries, MIBs and AIBs, but are the least mature. The compatibility of calcium metal anodes with electrolytes is an issue, with several known organic electrolytes causing either undesirable side reactions or the formation of insulating SEIs. An effective solution, such as organohaloaluminates for MIBs and ionic liquids for AIBs, has yet to be found for CIBs. Various transition metal chalcogenides and PBAs have been explored as potential cathodes, but charge capacities remain generally less than 100 mAh g^−1^, and cathode discovery remains an ongoing challenge [48, 121, 122]. Calcium is also slightly more expensive than sodium, aluminium, magnesium and zinc. These issues present CIBs with significant developmental roadblocks in the near future, especially in the presence of potentially cheaper, more mature and more energy-dense alternatives like MIBs and AIBs.

#### Dual-Ion Batteries

Unlike conventional ion batteries, which involve the insertion of the same ion into both the anode and cathode, dual-ion batteries (DIBs) involve the insertion of a cation into the anode and an anion into the cathode. This cation can be any of the aforementioned metal ions, while the anion is usually the counterion in the salt containing the cation, such as PF_6_^−^ or TFSI^−^. As the anodes insert cations, they are usually of the same design and composition as conventional ion batteries, and DIB efforts are mainly focused on the anion-inserting cathode [123–125].

It is notable that as the anode and cation are the same as in conventional ion batteries, DIB cost-savings must arise from the cathode. Among the earliest DIBs achieved was by the insertion of lithium salt anions into graphite cathodes. It avoided the use of expensive transition metal oxide cathodes and also had the additional benefit of potentially higher cell energy densities due to the higher cathode insertion potentials. However, these graphite cathodes suffered from very poor cyclability due to both the large volume expansion in association with the large anions, and the higher working voltages encouraging electrolyte decomposition. More recent efforts have thus been focused on novel cathodes such as various non-graphitic carbons, organic compounds and metal–organic frameworks [123–125]. While the cycle stability can be excellent, achieving several thousand cycles to 80% capacity [126, 127], voltages are generally reduced compared to graphite cathodes. This, in combination with generally low charge capacities, removes the potentially higher energy densities of DIBs. More importantly, most of these novel cathode materials are synthesised only at small scales and remain very expensive, making cost-effective commercialisation highly challenging.

## Addressing Capacity: Beyond Insertion Electrodes

### Limits of Insertion Electrodes

An obvious approach to increasing the energy density of an ion battery is to use insertion cathodes with higher potential or charge capacity. High voltage cathodes for LIBs have been a popular research topic over the past couple of decades as a result, most of which are based on polyanion oxides due to the inductive effect, such as LiCoPO_4_ having a discharge voltage against metallic lithium of 4.8 V [128], instead of ~ 4.3 V for LiCoO_2_. However, these polyanion oxides suffer from the same low conductivity issues as LiFePO_4_, sometimes to an even larger extent [129]. The increase in voltage also causes issues with electrolyte decomposition like in DIBs, leading to low cycle life, and the desired resultant higher energy capacity is partially offset by a decrease in theoretical charge capacity due to the inclusion of the additional anions. Spinel LiNi_0.5_Mn_1.5_O_4_ has shown the greatest promise among high voltage cathodes, with a discharge voltage of ~ 4.7 V against metallic lithium along with high electronic and ionic conductivity. However, it suffers the same Mn dissolution issues as spinel LiMn_2_O_4_ and an associated structural instability, leading to poor cycle life [130]. Higher charge capacity is achieved using lithium-rich layered oxides of the general form Li_x_MO_2_, where *x* > 1, but such cathodes suffer from voltage fade during charge/discharge for which the mechanism remains unclear, leading to poor cycle life [131].

**Fig. 10:**
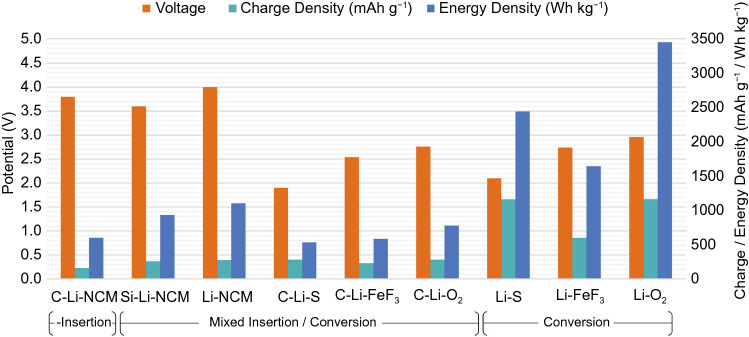
Theoretical charge capacity, voltage, and energy density of different lithium battery systems

Despite the promises and interest in high voltage and high charge capacity insertion cathodes, it is clear upon examination of the common LIB chemistries, C_6_-LiCoO_2_, C_6_-LiMn_2_O_4_ and C_6_-LiFePO_4_, that more than 90% of the electrodes’ mass arises from the insertion frameworks. As only a small proportion of these frameworks actively participate in the redox reaction, this places a limit on the theoretical charge capacities of insertion electrodes. The situation does not substantially improve even with highly lithium-rich systems such as C_6_-Li_2_MnO_3_. Instead, the next frontier in high-energy batteries is likely to lie in conversion electrodes. Unlike insertion electrodes, conversion electrodes do not require a framework to insert and remove active ions from, hence the proportion of active material in the electrodes can be close to 100%, leading to substantially higher charge capacities. Although the cell voltage is often lower, this is usually outweighed by the increase in charge capacity, leading to substantially higher energy densities overall (Fig. 10).

### Conversion Anodes

The high theoretical capacities of conversion anodes have attracted substantial research interest, and a large number of different anodes have been explored with several different mechanisms, including true conversion, alloying and active metal anodes. The so-called true conversion anodes (TCAs) are those that obey the most conventional definition of a conversion electrode, that is one that stores charge via cation replacement in an ionic compound by the active ion. Such systems are most commonly explored for alkali-ion batteries such as lithium and sodium, and the general reaction is depicted in Eq. 1, where *M* represents a cation, *X* represents an anion, and *A* represents the active ion [132].

1 $${M_\alpha }{X_\beta }\; + \;\beta n \cdot {A^ + }\; + \;n \cdot {e^ - }\; \leftrightarrow \;\alpha \cdot M\; + \;\beta \cdot {A_n}X$$

A very large number of possible compounds have been explored, including various transition metal oxides, sulphides, nitrides and phosphides [132–134]. However, TCAs typically suffer from poor reversibility, high charge/discharge voltage hysteresis and low current capabilities, arising from poor electronic conductivity of both the original *M*_*α*_*X*_*β*_ and *β*·*A*_*n*_*X* reaction product [135]. In addition, the average reaction potentials often exceed 1.0 V against metallic lithium, reducing the achievable cell potentials and limiting the cell energy density. Both practical and theoretical capacities are also generally limited to the region of 1000 mAh g^−1^ or less. This, while substantially higher than insertion anodes, pales in comparison to the several thousand achievable by alloying and active metal anodes, which will be the main discussion focus of Sect. 4.2.

Also of note are anodes with both insertion and conversion mechanisms, such as graphite intercalation compounds (GICs) [136]. For example, transition metal chloride-intercalated graphite has been shown to store lithium first through intercalation into the remaining space between graphite sheets, followed by a conversion reaction with the pre-intercalated metal chloride according to Eq. 1. In this case, the intercalation and conversion species are symbiotic. Presence of the metal chloride between graphite sheets expands the interlayer spacing, increasing ionic conductivity, while the graphite enhances the electronic conductivity of the metal chloride and provides a buffer matrix for the volume expansion during lithiation [137]. Nonetheless, GIC reversible charge capacities remain in the region of 1000 mAh g^−1^ for lithium, and reaction potentials remain fairly high compared to metallic lithium. Hence, like TCAs, they appear to be less attractive than the high capacity, low potential alloying and active metal anodes.

#### Alloying Anodes

Alloying anodes are similar to insertion anodes in that they both store charge in the form of active ions, but a phase change usually occurs in the former that allows a substantially higher ion capacity. However, this higher ion capacity coincides and correlates with a substantially larger volume expansion during storage, which may lead to disintegration of the anode during cycling [138]. While a large number of different alloying anodes have been explored for various active ions, the issues faced such as the aforementioned volume expansion are often very similar and will be presently illustrated in by far the most popular example, lithium-silicon.

Silicon is known to be the highest energy alloying anode for lithium due to its very high charge capacity of 2009 mAh g^−1^ for the most highly lithiated Li_22_Si_5_ phase, including lithium mass, coupled with a redox potential of ~ 0.4 V against metallic lithium, allowing for high cell potentials. While the low electronic and lithium ionic conductivity is an issue, the main barrier to commercial adoption is the extremely high volume expansion of ~ 400% during lithiation [139], 40 times that of graphite. This enormous volume change during cycling can cause fracturing of the silicon anode, at first disrupting electronic conduction and eventually leading to disintegration of the entire anode. Fracturing of the SEI also occurs, resulting in continuous conversion of active lithium to inactive compounds and electrolyte consumption due to the re-formation of the SEI. Both of these effects lead to very poor cyclability of the anode [140, 141].

**Fig. 11:**
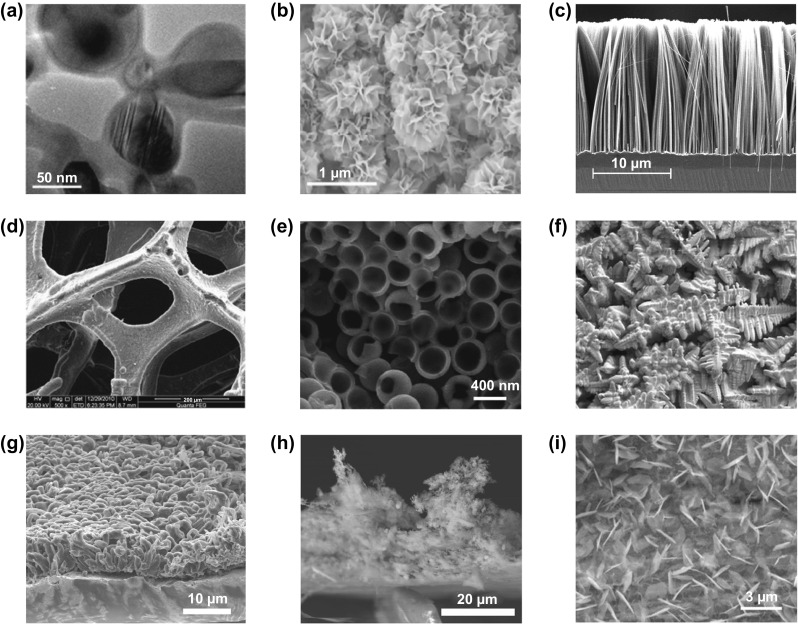
Scanning electron micrographs showing various, selected nanostructured silicon anodes. **a** core–shell silicon-carbon nanoparticles [147]. Copyright 2012, Elsevier. **b** Spongy nanographene encapsulating silicon nanoparticles [149]*.* Copyright 2017, Springer Nature. **c** Aligned silicon nanowires [152]*.* Copyright 2008, AIP Publishing. **d** Silicon thin film deposited on nickel foam [153]*.* Copyright 2012, Elsevier. **e** 3D porous anode formed from hollow silicon nanospheres permission [154]*.* Copyright 2011, American Chemical Society. Scanning electron micrographs showing various dendrite morphologies. **f** Melt-grown crystal dendrites [155]*.* Creative Commons License (CC BY). **g** Electrochemically grown lithium dendrites [156]*.* Copyright 2016, American Chemical Society. **h** Electrochemically grown aluminium dendrites [157]. Copyright 2017*,* American Chemical Society. **i** Electrochemically grown zinc dendrites [158]*.* Copyright 2020, John Wiley and Sons

The general strategy to address volume expansion is to reduce feature sizes of the silicon anode, as smaller structures generally show lower tendencies to be damaged when subjected to mechanical stress [142, 143]. Conveniently, this simultaneously addresses the conductivity issue as diffusion lengths are reduced. 0D nanostructures in the form of nanoparticles require a conducting matrix that physically and electronically anchors them to the current collector. Carbon is a popular matrix due to its simultaneous ability to store lithium ions, forming silicon carbon composite anodes. The matrix also buffers the volume expansion of the anode as a whole, aiding in the structural preservation of the SEI during cycling [144]. While addition of small amounts of silicon to primarily graphite matrices is already sufficient to substantially improve charge capacities [145, 146], the usage of more complex structures like core-shell nanoparticles [147] **(**Fig. 11a) or the anchoring of silicon to highly conductive graphene scaffolds [148, 149] (Fig. 11b) can allow charge capacities much closer to theoretical limits.

1D, 2D and 3D nanostructured silicons have all been explored extensively as means to reduce feature sizes. For example, 1D structures in the form of nanowire forests (Fig. 11c) can resist cracking due to the relative ease of strain relaxation inherent in their morphology [150]. Adopting nanotubes instead of nanowires can further improve performance due to their hollow structure allowing more room for volume expansion, as well as a greater surface area for electrolyte access. 2D structures in the form of thin films (Fig. 11d) likewise resist cracking, with mechanical stabilisation arising from good adhesion to the current collector substrate. However, the effect only persists over film thicknesses on the order of one micron or less [140]. Hence, increasing film thickness requires the incorporation of pores to provide space for volume expansion, forming 3D porous nanostructures (Fig. 11e). Nonetheless, while these approaches are effective in preserving the structural integrity of the silicon, they do not address the SEI fracturing issue, which is tackled by the simultaneous addition of carbon coatings or matrices, as is the case with silicon nanoparticles.

Generally, the strategy of reducing feature size in silicon anodes has been fairly successful for both nanoparticulate and nanostructured anodes. Charge capacities routinely fall within the range of 1000–3000 mAh g^−1^ (excluding lithium mass), accompanied by cycle lives of several hundred [140, 141]. Remaining challenges lie in the translation of laboratory-scale set-ups to practical commercial cells instead. While nanostructured anodes generally achieve higher charge capacities than nanoparticulate anodes due to higher silicon content, fabrication methods are also generally far more complicated and expensive. Nanostructured anodes, especially in 1D and 2D forms, also suffer from low areal mass loading, resulting in low practical cell capacity as the anode mass becomes dominated by the current collector. Nanoparticulate anodes thus remain popular, especially from the perspective of industrial adoption [144, 151].

#### Active Metal Anodes

Active metal anodes have the highest theoretical energy densities for each cationic species, as they consist of 100% active material, and have been in use for far longer than insertion or alloying anodes in primary batteries. However, recharging remains a major challenge despite decades of research and development. Reactions with the electrolyte is a common issue, causing corrosion of the anode, decomposition of the electrolyte or the formation of passivating SEIs. The exact causes and consequences of these reactions differ greatly among different metals, as has been covered in the earlier section on alternative insertion ions for various multivalent metal species, and the general solution is to alter the electrolyte composition.

The more challenging barrier for rechargeable metal anodes is the tendency for non-uniform plating during the charging process, usually in the form of high aspect ratio protrusions that are commonly referred to as dendrites. It should be noted that despite the name, metal anode dendrites share very little similarities with the more well-known crystal dendrites formed during melt solidification, and can exhibit various different morphologies depending on the metal and electrolyte combination (Fig. 11f–i). In many cases, they are not even strictly dendritic, but are nonetheless referred to as dendrites [158]. Nonetheless, regardless of morphology, the effect on cell performance is similar. Uncontrolled dendritic growth over repeated charge/discharge cycles can eventually cause piercing of the separator and physical contact between the anode and cathode, short-circuiting the cell. Repeated non-uniform dissolution and growth at the anode can also gradually reduce the amount of anodic active material due to dendrites physically breaking off, or the formation of insulating SEI-coated deposits [159]. Both of these effects are responsible for the characteristically short cycle life of metal anodes. Most investigations into dendritic growth mechanisms and solutions are focused on zinc and lithium anodes in aqueous and organic electrolytes, respectively, due to their relative maturity at the cell level. However, the general strategies to address dendritic growth are fairly similar, and can be carried over to other multivalent aqueous and monovalent organic systems.

The general mechanism of dendritic growth occurs via initial nucleation, occurring due to inhomogeneities on the anode surface or regions of damaged SEI, followed by self-promoting growth due to the intensification of the local electric field at dendrite tips facilitating further metal deposition on the tips [160, 161]. Strategies against dendritic growth can tackle the issue at either the nucleation or growth stage. A common approach is to deposit an artificial SEI ex-situ, that is more stable that the SEI that would normally form, on the surface of the anode. This artificial SEI is usually designed to be ionically conductive, allowing ions to pass through and deposit in-between itself and the current collector, but suppressing dendritic growth via physical confinement. Ionically insulating artificial SEIs can also be used, which encourage uniform plating via the provision of uniformly distributed nucleation sites rather than physical confinement, although the effect is limited to thin layers of metallic lithium which restricts active material loading [158, 162, 163].

Nanostructured current collectors, such as porous copper or carbon scaffolds, is another possible approach [158, 162, 163]. Dendritic growth is addressed by confining them to within the scaffold, where they are less likely to grow towards the cathode and more likely to maintain electrical contact with the current collector after structural damage. Scaffolds can be combined with artificial SEIs to further control dendritic growth within the scaffold. In the case of ionically insulating SEIs, the scaffold also provides a greater surface area for active material loading. However, this approach dramatically increases the cost and weight of the current collector, negatively affecting performance at the practical cell level.

Electrolytes can also be modified to control dendritic growth. Reactive additives can be used to facilitate the in-situ generation of SEIs that play the same role as the aforementioned artificial SEIs. On the other hand, highly polar non-reactive additives can selectively adsorb themselves onto dendrite tips due to the intensified electric field, shielding them from the active cations and disrupting the self-promoting growth mechanism [158, 162, 163]. A more direct approach is to use solid electrolytes that mechanically suppress dendritic growth. However, challenges remain with this approach as well, as the effectiveness of suppression is dependent on the stiffness of the electrolyte, and dendritic growth is still possible through defects in the solid electrolyte such as voids and grain boundaries [164]. Solid electrolytes also face their own set of issues, such as poor interfacial stability with the electrode, that need to be separately addressed.

In general, the various strategies for suppressing dendritic growth have shown successful suppression over several hundred cycles [158, 162, 163]. However, the effect on cell-level performance is poorly documented. This is as failure due to dendritic growth is highly dependent on the cell geometry, and cell-level performance recorded in typical laboratory cells, which generally have physically further separated electrodes than tightly packed commercial cells, is not very useful in predicting the performance of the latter.

### Conversion Cathodes

Negative ions occupy far less space on the periodic table than positive ions, reflecting a smaller selection of elements, concentrated in Groups 5, 6 and 7, to choose from. This is further complicated by the fact that most light anionic elements are gaseous at around room temperature. By far the most popular conversion cathodes are based on the light chalcogens, sulphur and oxygen, although halogen cathodes also exist. Pnictogens, despite high theoretical charge capacities, are generally too stable to be used as conversion cathodes at normal battery operating temperatures.

### Halide Cathodes

Fluorine is an especially attractive element due to its very high standard reduction potential of + 2.87 V coupled with a high charge capacity of 1409 mAh g^−1^ for the F^-^ ion, leading to very high theoretical energy density. However, fluorine is a toxic gas at room temperature, a property of nearly all stable halogens except iodine, which has an energy density less than 10% of fluorine against the SHE due to its low redox potential and charge capacity. Halide cathodes are thus usually made solid by utilising them as metal halides, with transition metal fluorides being the most popular.

**Fig. 12:**
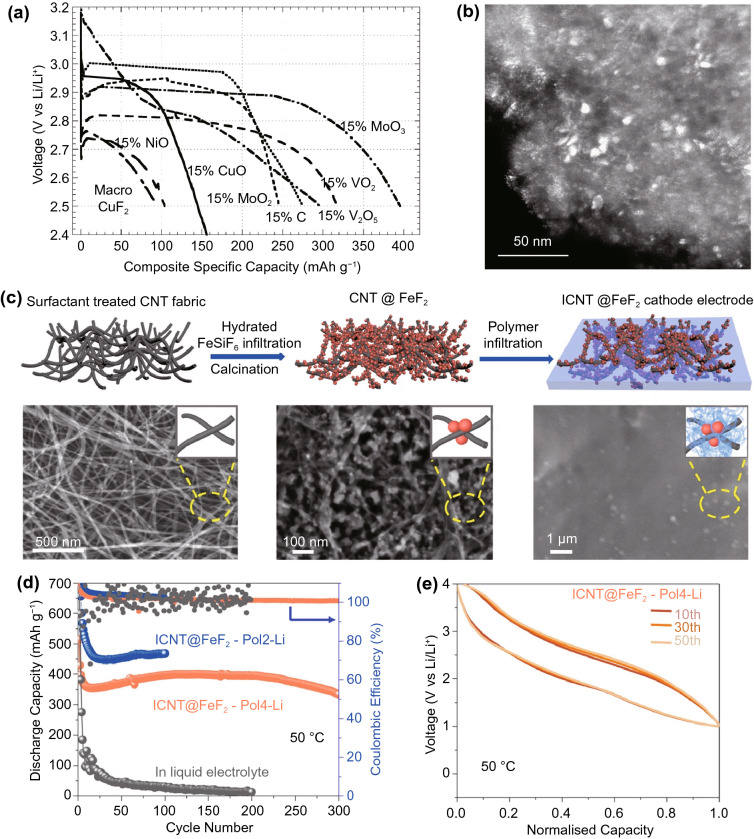
**a** Discharge capacity of CuF_2_ composite cathodes with different conductive matrices_,_ and **b** a transmission electron micrograph depicting CuF_2_ nanoparticles within a conductive matrix [167]*.* Copyright 2007, American Chemical Society. **c** Schematic of a FeF_2_ cathode on a conductive carbon nanotube scaffold that provides good electronic access, while the polymer electrolyte controls dissolution, leading to a **d** high capacity, **e** high cycle life cathode [170]*.* Copyright 2019, Springer Nature

While the incorporation of the inactive transition metal reduces both the charge capacity and redox potential of fluorine, theoretical energy density remains high due to the high ionicity of the fluoride bond leading to generally higher redox potentials than other solid conversion cathodes. However, like TCAs, the same high ionicity also leads to very low electronic and ionic conductivity of both the transition metal fluoride and the conversion reaction product, such as lithium fluoride, in the case of pairing with a lithium anode [14, 25]. Aside from causing generally high overpotentials and low current capabilities [165], this also results in generally poor practical capacities due to rapid passivation of the cathode during discharge, requiring dispersion as nanoparticles in conductive matrices to resolve (Fig. 12a, b) [166, 167].

The discharge products of transition metal fluoride cathodes are the anodic metal fluoride and the transition metal. This substantially complicates recharging as the charging reaction involves two species that have to be kept in electrical contact with the current collector in order to regenerate the transition metal fluoride and anodic metal. CuF_2_ cathodes paired with lithium anodes had received ample early attention due to the high energy density enabled by a remarkably high potential of ~ 3.55 V against metallic lithium [14]. However, recharging is very difficult due to the tendency for the copper reaction product to agglomerate into nanoparticles insulated from the current collector by a matrix of lithium fluoride [168]. FeF_3_ has similar theoretical energy density to CuF_2_, but suffers less from this issue due to the lower diffusivity of the higher charge density Fe^3+^ ion, facilitating the formation of percolating conductive iron networks upon discharge [169]. However, reversibility remains generally limited. This, in combination with the generally high solubility of transition metal halides in common organic electrolytes [165] and their tendency to form structurally unstable SEIs [170], results in generally very low cycle lives.

Furthermore, the FeF_3_ reaction occurs via a complex mechanism involving both insertion and conversion. The latter is responsible for most of the charge capacity, but is disabled by the absorption of water, which occurs very easily due to the generally high hygroscopicity of metal fluorides. This complicates fabrication processes for batteries hoping to utilise the conversion reaction, even at the laboratory scale [171]. Despite these challenges, some encouraging results of around 1000 Wh kg^−1^ (Fig. 12c–e) cathodic energy densities with cycle lives of 300–1000 have been achieved [170, 172]. Nonetheless, the poorer theoretical energy density of metal halides compared to the elemental chalcogens contribute to generally lower research interest. As a result, overall progress is substantially slower than the chalcogens.

#### Sulphur Cathodes

Sulphur and phosphorus are the two lightest solid anionic elements in the periodic table; however, the latter generally cannot undergo conversion reactions with metallic cations at around room temperature, and has instead been investigated for use as an insertion anode [19]. Hence, sulphur can boast the highest charge capacity among solid conversion cathodes, at 1670 mAh g^−1^ for the S^2−^ ion. Although the main discharge plateau occurs at ~ 2.1 V against metallic lithium, substantially lower than in transition metal fluorides, the very high charge capacity allows sulphur to have the highest theoretical energy density among all solid conversion cathodes of ~ 2500 Wh kg^−1^ in the form of Li_2_S. Sulphur is also extremely cheap. While sulphur cathodes can be paired with a large number of different cationic species [17], the challenges faced at the cathode are largely similar. By far the highest capacities have been achieved in and the greatest amount of research has been focused on the lithium–sulphur couple, which is the archetypal system used presently to discuss sulphur cathodes.

The challenges faced by sulphur cathodes are somewhat similar to other solid conversion electrode materials [173–175], including a large volume expansion of ~ 80% upon discharge leading to structural damage and very low electronic and ionic conductivity. Active material dissolution is also an issue, like metal fluorides, but through a different mechanism. Elemental S_8_ is gradually reduced to S^2-^ during the conversion through S_8_^2−^, S_6_^2−^, S_4_^2−^ and S_2_^2−^ polysulphide intermediates. While S_2_^2−^ and S^2−^ form insoluble Li_2_S_2_ and Li_2_S when paired with lithium, and S_8_ itself is also insoluble, the longer chain polysulphide compounds are soluble in typical organic solvents. These soluble polysulphides can be beneficial to cell performance by aiding uniform sulphur dispersion in the cathode and acting as redox mediators [176, 177], but they also diffuse to the anode and form short-circuited lithium–sulphur couples, generating insoluble Li_2_S and leading to active material loss from the cathode. Although some of this Li_2_S can be oxidised back to soluble polysulphides during charging and redeposited at the cathode, this often leads to morphological changes that generate insulated, inactive sulphur. This so-called polysulphide shuttle effect is responsible for the main practical challenge faced by sulphur cathodes, appreciable self-discharge and a generally low cycle life.

**Fig. 13:**
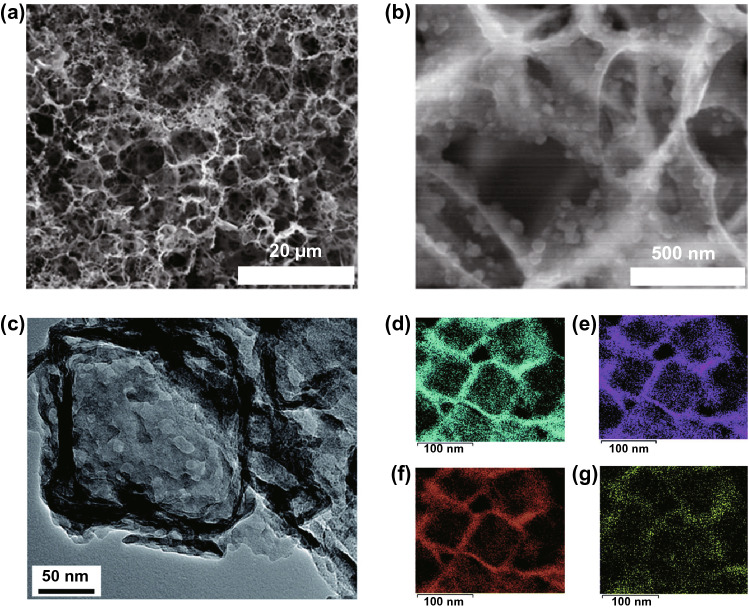
Scanning electron micrographs of a 3D porous carbon scaffold onto which sulphur nanoparticles are deposited, at **a** low magnification and **b** high magnification [180]. Creative Commons License (CC BY 4.0). **c** Transmission electron micrograph of a polysulphide-adsorbing TiO_2_-MnO nanobox cathode infiltrated with sulphur, and associated energy-dispersive X-ray spectroscopy maps for **d** Ti, **e** Mn, **f** O and **g** S [181]*.* Copyright 2021, Royal Society of Chemistry

Like silicon, the main approach adopted to address the conductivity and volume expansion issues of sulphur is by reducing feature sizes. However, rather than being grown directly as nanostructures, a porous scaffold is usually synthesised instead, followed by infiltration of molten sulphur. Carbon scaffolds are popular in a variety of different forms, including nanotubes, graphene, nanofibers and porous 3D carbon (Fig. 13a, b). Polysulphide shuttle in carbon is generally addressed simply by more tortuous diffusion paths out of the scaffold, which has shown encouraging results in laboratory environments, where cycling rates exceed the time scale at which polysulphide diffusion occurs. This may not be the case in practical cells as they undergo intermittent use accompanied by long storage times, resulting in more stringent requirements on polysulphide diffusion rates and echoing similar challenges in LIB testing [178]. In the case of extremely small pores of less than 1 nm, polysulphide shuttle can also be reduced by sterically preventing the formation of the soluble long-chain polysulphides [179]. Carbon-scaffold sulphur cathodes are generally able to achieve charge capacities of around 1000 mAh g^−1^ with cycle lives in the region of 100–300 [17, 173, 174].

A more sophisticated approach of preventing polysulphide shuttle involves using scaffolds that are able to chemically coordinate with the soluble polysulphides through polar interactions (Fig. 13c–g), which has been observed in a variety of compounds including chalcogenides, oxides, nitrides and organic compounds. This can also be achieved on carbon scaffolds through the addition of heteroatoms. The coordination strength between such scaffolds and the soluble polysulphides must be finely controlled so that it is strong enough for good adsorption, yet not so strong that it is favoured over the lithium–sulphur bond, which would make the sulphide inactive. Such compounds also generally have lower conductivities, so a portion of the scaffold often remains carbon-based. Nonetheless, cathode scaffolds based on polysulphide coordination are generally able to achieve similar charge capacities as carbon scaffolds of around 1000 mAh g^−1^, with slightly improved cycle lives in the region of 200–500 [17, 174].

A separate approach involves the elimination of the soluble polysulphides from the conversion process with catalysts, nonetheless deposited on porous scaffolds, that can facilitate fast reactions directly to the solid sulphides. The catalytic mechanism relies on strong adsorption of long-chain polysulphides, while maintaining a sufficiently high sulphide diffusion coefficient for efficient surface nucleation and growth of the solid sulphides. High electronic conductivity is also important for electrons to access the insulating sulphides [182]. A large number of catalysts have been explored including noble metals, chalcogenides, nitrides, various metal-free materials, as well as heterostructure combinations of these materials. It is notable that many of these are also used in the desulphurising industry [183]. While the performance of catalysed lithium–sulphur batteries is highly variable, remarkable results of around 80% capacity retention at 2000 cycles with a 600–800 mAh g^−1^ initial capacity have been achieved with TiO_2_-TiN heterostructures [184].

Other than addressing the issue at the cathode site, polysulphide shuttle can also be tackled at the cell level by the usage of ion-selective separators that allow active ion transmission but block the larger polysulphide ions. The mechanisms can be physical filtration or chemical adsorption, similar to those in nanostructured cathodes, but also electrostatic repulsion, taking advantage of the different charges of the negative polysulphide and positive active ions [185]. However, the results achieved with this approach are not significantly better than the others, with 80% capacity retention generally only possible for a few hundred cycles [185–187], and like catalysts, does not remove the need for nanostructured cathodes to address conductivity and volume expansion issues.

Despite the steady progress made over recent years in addressing the various issues associated with sulphur cathodes leading to poor cycle life, considerable challenges nonetheless remain in the translation of laboratory-scale set-ups to practical cells. Lithium–sulphur cell performance is affected by the volume of electrolyte available, which needs to be minimised in practical cells. Lower electrolyte volume can lead to higher soluble polysulphide concentrations, reducing ionic conductivity and suppressing further formation of soluble polysulphides at the cathode, which is a necessary part of the discharge process [188]. Similar to silicon, the various nanostructuring approaches adopted to control cyclability issues also generally lead to high fabrication costs and low active sulphur loading in the cathode, which limits the capacity of a practical cell [174, 175].

#### Oxygen Cathodes

Oxygen has an even higher charge capacity than sulphur due to the lower atomic mass, and in combination with generally higher redox potentials, gives it one of the highest possible theoretical energy densities among all cathodes, exceeding 4000 Wh kg^–1^ for the simple oxides of aluminium, magnesium and lithium [15]. Oxygen is also unique among gaseous electrodes due to its high reactivity and natural abundance in the atmosphere, rendering oxidation to be the preferred redox reaction when cationic elements are exposed to air. This allows oxygen cathodes to be used as air cathodes, extracting the gaseous active species from the environment rather than having to carry them around in expensive and heavy high-pressure vessels. It should be noted that the ability to extract oxygen from the atmosphere does not mean that the cathode active species mass can be neglected, as the oxygen still needs to be carried within the cell in the form of the oxide reaction products, especially if the battery is to be rechargeable. As a result, an oxygen cathode cell will display the unusual characteristic of becoming heavier as it discharges, and lighter as it charges.

The oxide reaction product, and thus both the energy density and rechargeability are heavily dependent on the electrolyte. In aprotic organic solvents, lithium tends to form the solid Li_2_O_2_ peroxide, sometimes through a LiO_2_ superoxide intermediate that reacts with the electrolyte to form the peroxide [189]. In an aqueous solvent, the reaction product is the Li^+^ ion and water in acidic environments, and soluble LiOH in basic environments. The cell voltage also varies, due to the pH-dependence of the oxygen reduction reaction [190]. Magnesium and aluminium likewise form hydroxides in aqueous environments, which are so stable that the cells are considered practically non-rechargeable. Rechargeability is only observed in non-aqueous electrolytes, where the simple oxides tend to be formed [191, 192]. Zinc, on the other hand, tends to form the ZnO simple oxide in aqueous environments, which is relatively easier to recharge compared to magnesium and aluminium [193]. Hence, a significant challenge in oxygen cathode cell design is the selection of a suitable electrolyte. It should be noted that the anode is usually in metallic form as usage of an alloying or insertion anode requires a cation source, leading to the well-known metal–air battery and further complicating electrolyte selection due to the simultaneous need to form stable and conducting metal–electrolyte interfaces.

Despite the differences in preferred reactions, a major and common challenge that all oxygen cathode cells face is the slow reaction kinetics of the oxygen reduction (ORR) and oxygen evolution reactions (OER), occurring at the triple phase boundary between the gaseous oxygen, solid catalyst and liquid electrolyte, leading to poor current capabilities of the cell. For the past two decades, a significant portion of the research effort on oxygen cathodes has thus been dedicated to the development of more efficient ORR and OER electrocatalysts, with the general goals of increasing power density and cycle life, and reducing the charge/discharge overpotential. High-performance catalysts for the ORR and OER are conventionally based on platinum group metals (PGMs) and their alloys, due to their favourable binding energies with reaction intermediates [194]. However, their scarcity and high cost have prevented their widespread use, and catalyst developments are generally focused on matching or exceeding the performance of PGMs with lower cost alternatives.

**Fig. 14:**
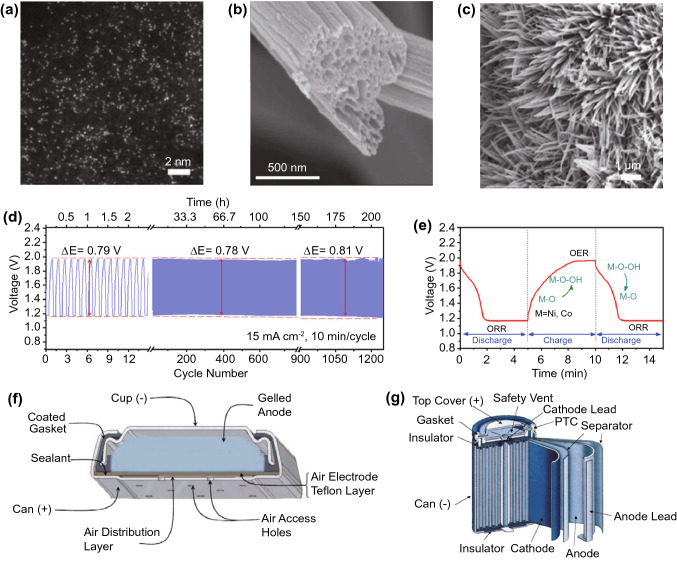
**a** Transmission and **b**, **c** scanning electron micrographs of various air cathode catalyst structures. **a** Pt single atoms on a graphdiyne substrate [208]*.* Copyright 2018, John Wiley and Sons. **b** porous nitrogen-doped carbon fibre [204]*.* Copyright 2013, Elsevier. **c** Co_3_O_4_ nanowire core and NiFe-layered double hydroxide shell (LDH) structures. Electrochemical performance of the Co3O4@NiFe LDH catalyst, showing **d** high cycle stability but **e** the characteristically low round trip efficiency caused by the large potential gap between the ORR and OER [209]*.* Copyright 2019, American Chemical Society. **f** Schematic of a typical zinc–air prismatic cell, showing a planar electrode configuration [210]*.* Copyright, Energizer Battery Manufacturing. **g** Schematic of a typical cylindrical LIB, showing a coiled electrode configuration [211]*.* Copyright 2013, Macrothink Institute. The coiled electrodes offer substantially a larger surface area in the same volume

A large variety of alternative catalysts have been studied for the ORR, most of which are based on transition metal compounds and carbon (Fig. 14a–c) [195, 196]. The surface binding energy between the catalyst and reaction intermediates is one of the key determinants of catalyst performance. Hence, modulation of the electronic structure through strategies such as alloying or crystal structure control, as well as lattice defect engineering have been invaluable tools in improving the ORR performance of transition metal oxide and nitride catalysts. On the other hand, catalytic activity in carbon compounds is improved by doping of heteroatoms such as nitrogen-coordinated metals, forming the popular metal–nitrogen–carbon (M–N–C) class of catalysts. Defect engineering can likewise be employed, usually with the aim of increasing the concentration of more catalytically active edge carbon atoms. The usage of nanoclusters or single atoms of PGMs has also enjoyed high recent popularity [197, 198], as they both increase catalytic activity per unit mass of PGM and decreases the mass loading of the catalyst, potentially sidestepping their high cost. However, the lower mass loading is a double-edged sword, as although the activity per unit mass of the catalyst may be improved, overall electrode performance can become worse if this improvement is insufficient to outweigh the decrease in the amount of catalyst available. Strategies for improving OER catalysts are largely similar to the ORR [199, 200]. Also important for rechargeable oxygen cathodes is the development of bifunctional catalysts that can catalyse both the OER and ORR. While separate catalysts can be held on the same support for these two reactions, most of them are degraded during charge/discharge due to the alternating reductive and oxidative environments of the ORR and OER, respectively [201]. Design of these bifunctional catalysts again utilises similar strategies to ORR and OER catalysts [202, 203].

In general, these strategies have resulted in bifunctional catalysts in zinc–air batteries with peak power densities of around 200–300 mW cm^−2^ [201, 203], capable of matching commercial Pt/C catalysts under similar conditions [204, 205]. This performance also exceeds those of MnO_2_ catalysts that are more commonly used for commercial zinc–air batteries, which are typically around 100 mW cm^−2^ [206]. However, high charge/discharge overpotentials of around the same values as the ORR potential leads to round-trip efficiencies of only around 50% (Fig. 14d–e). Primary aluminium–air batteries are likewise able to achieve power densities of around 200 mW cm^−2^ [192]. However, these high power densities are usually achieved during galvanodynamic testing. Galvanostatic cycling tests are typically done at more modest power densities on the order of 10 mW cm^−2^, similar to the figures commonly observed for lithium–air batteries [189, 207], and it is unclear if the galvanodynamic power densities can be sustained under typical battery discharge conditions.

Besides the issues with OER and ORR kinetics, oxygen cathodes also face several practical challenges arising from the need for the cathode to be exposed to the atmosphere. In the case of LIBs, current capabilities at the cell level can be easily increased by increasing the electrode area through coiled designs, albeit at the cost of energy density due to increasing current collector mass. However, doing the same for oxygen cathodes is much more complicated due to the need for sufficient airflow to the cathode (Fig. 14f–g). Improving power density by improving accessibility of the cathode to ambient air also facilitates electrolyte contamination and loss through evaporation, which must be mitigated with selective membranes [189]. Solid oxide reaction products, such as Li_2_O_2_, are also usually insulating, causing them to gradually cover and deactivate the catalyst active sites. This leads to the practical capacity of the battery scaling with electrode surface area rather than the mass of metal available at the anode [212]. Soluble reaction products, such as LiOH, face the separate issue of a finite solubility limit in the electrolyte, again leading to the practical capacity scaling not with the anode mass, but with electrolyte volume instead.

## Addressing Safety: Beyond Organic Liquid Electrolytes

While the exact electrolyte compositions are principally dependent on the electrode selection, the majority of high-energy battery systems, including LIBs, use organic solvents into which a salt is dissolved as the ion source. The dissolved ions provide sufficiently high ionic conductivity, while the organic solvent provides a sufficiently large electrochemical stability window (ESW) for the battery to function. However, most of these liquid organic electrolytes are highly flammable, driving the desire to eliminate their usage in order to improve safety.

One solution is to utilise flame-retardant additives in the electrolyte, which generally function by scavenging the oxygen and hydrogen radicals responsible for the propagation of combustion reactions [213–215]. Several different additives have been explored, the most common being phosphate- and fluoride-based compounds. While these can indeed be effective in reducing the flammability, the generally high concentrations required of < 20% increases the electrolyte viscosity and reduces ionic conductivity. The additives may also react with the electrodes or decompose at high potentials, reducing the cell cycle life. Raising the salt concentration in the electrolyte can also reduce the flammability by reducing the amount of free solvent molecules, but comes with higher costs due to the quantity of salt required [213–215].

Nonetheless, despite significantly improved safety, organic electrolytes with flame-retardant additives remain inherently flammable, and their total replacement with aqueous or solid electrolytes is considered a more complete solution to the problem. It should be noted that substitution of solely the electrolyte usually cannot increase the energy density of the battery, as the active material does not change. However, electrolyte substitution sometimes enables the use of different cell designs which can improve energy density, such as solid electrolytes enabling the use of metal conversion anodes and eliminating the separator.

### Aqueous Electrolytes

**Fig. 15:**
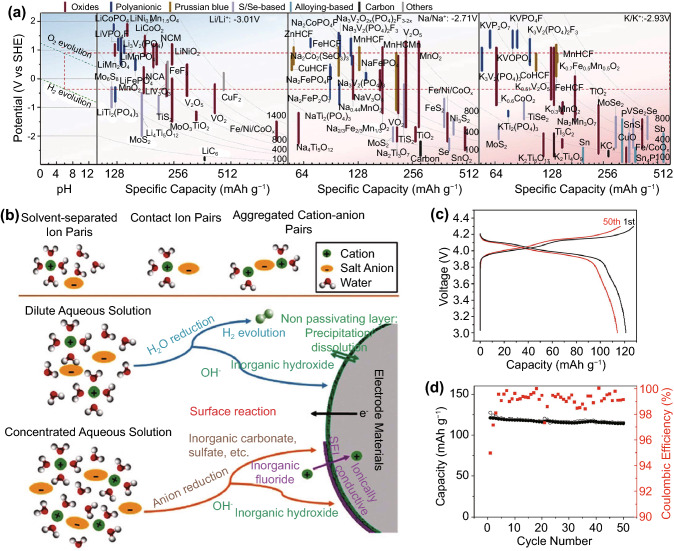
**a** Illustration of the position of the ESW of water relative to common anodes and cathodes for lithium and sodium ions [222]. Creative Commons License (CC BY-NC 4.0). **b** Illustration of some solvated ion-water interactions, and the shift from water to anion reduction in response to an increase in salt concentration [220]*.* Copyright 2020, John Wiley and Sons. **c** High voltage and **d** Coulombic efficiency achieved by a combined WiSE and artificial SEI approach, showing expansion of the ESW to above 4 V for LIBs [223]*.* Copyright 2017, Elsevier

Aqueous electrolytes, consisting of an active ion-containing salt dissolved in water, are a safe alternative to the widely used organic electrolytes due to their inherent non-flammability. However, the perennial issue that they face is the electrolytic decomposition of water. This occurs via the OER at the cathode and hydrogen evolution reaction (HER) at the anode, respectively, with a stable voltage window between them of only 1.23 V for pure water. Aside from depletion of the electrolyte and corrosion of the electrodes, both leading to low cycle life, the evolution of hydrogen gas also leads to potential safety issues due to its high flammability [216]. The small ESW, aside from lowering cell potential, may also limit the electrode selection to lower charge capacity options, such as VO_2_ instead of carbon for lithium anodes (Fig. 15a) [217]. Both effects severely limit overall cell energy density.

Suppression of water decomposition is usually done kinetically, with the effect of increasing the overpotential of the reaction. One approach is to design appropriate competing reactions that occur near the potentials at which water splitting occurs, but with the former favoured due to faster reaction kinetics. This can be achieved by both electrode modifications [218] and electrolyte additives [219]. However, the stability enhancements achieved using this method are usually fairly modest.

A more successful strategy is to make use of the tendency for the ESW to become larger as salt concentration in the electrolytes increase [220]. This is as the ions present in solution are coordinated by a shell of water molecules, and increasing the ion concentration decreases the amount of free water available for the decomposition reaction. This, again, is a form of kinetic control, and promotes other, less harmful reactions instead of water decomposition (Fig. 15b). Very large stability windows of 2–3 V can be achieved by increasing the salt concentration to the extent that there are very few free water molecules remaining, known as water-in-salt electrolytes (WiSE) [221]. The higher salt concentration also facilitates the formation of protective SEIs on the electrodes, and the approach has been demonstrated with various different active ions including sodium, zinc, magnesium and aluminium.

However, 2–3 V remains insufficient for most LIB and SIB chemistries, and it is challenging to make aluminium and magnesium anodes compatible with aqueous electrolytes for the reasons discussed in Section 0. The WiSE stabilisation effect is also generally more effective at the cathode, given the tendency for battery cathode materials to be closer to the OER potential than anode materials are to the HER potential (Fig. 15a) [222]. Thus stability at the anode remains a challenge, for which the general solution is to use an artificial SEI. The largest ESW reported to date exceeds 4 V, comparable to the stability window for organic electrolytes, and was demonstrated through the combined application of an artificial SEI and WiSE in an LIB (Fig. 15c, d) [223]. The large ESW enabled the use of conventional graphite anodes, and in combination with transition metal oxide cathodes, resulted in cell energy densities that are no worse than organic LIBs. However, the cycle life of 50 remains too low for practical applications, which was attributed to insufficient quality and stability of the anode SEI. Hence, better artificial SEIs need to be found before aqueous high-energy batteries can compete with their organic counterparts.

Solid electrolytes can be inorganic (e.g. ceramics and glasses), organic (e.g. polymers) or a composite, which is simply a combination of the two. Their popularity stems from their potential ability to simultaneously address several issues with high-energy batteries. Polymer solid electrolytes generally have lower flammability than liquid organic electrolytes, and inorganic solid electrolytes are often completely non-flammable. At the same time, their high stiffness can prevent the formation of dendrites for metallic anodes and remove the need for a separator, while the lack of any liquid medium also resolves the dissolution issues faced in several conversion cathodes. While solid electrolytes have been explored in a variety of active ion systems, the vast majority of work is focused on lithium, and relatively few studies have been reported for multivalent ions such as aluminium, magnesium and zinc. Conductivity in inorganic electrolytes is generally very low at room temperature for such ions due to their low ionic mobility, and most solid electrolytes are polymer-based [74, 92, 224, 225]. Sodium has received slightly more attention, but the systems investigated are generally fairly similar to their lithium analogues [49, 226]. The present discussion will thus focus on lithium-ion solid electrolytes.

#### Inorganic Solid Electrolytes

Ionic conduction in inorganic solid electrolytes occurs via the movement ions through diffusion pathways in the crystalline lattice, or energetically favourable local sites in amorphous structures. The design of inorganic solid electrolytes thus shares some similarities to insertion electrodes, being generally a lithium-containing compound with facile diffusion pathways. However, two important differences are that in the case of electrolytes, electronic conductivity is to be minimised instead of maximised, and ionic conductivity is more important than storage capacity.

**Fig. 16:**
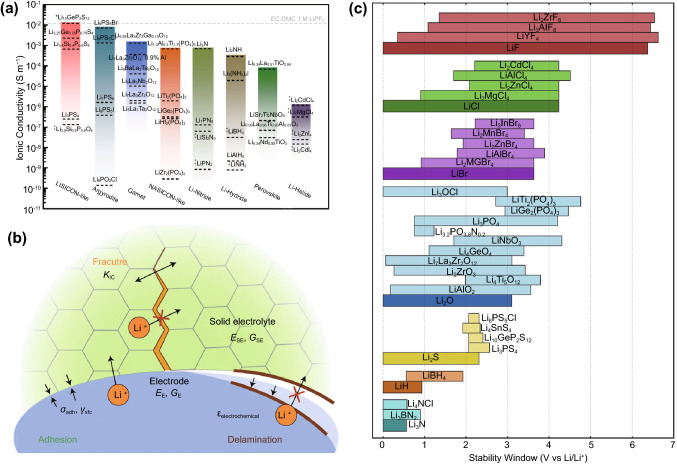
**a** Lithium ionic conductivity of selected inorganic solid electrolytes and a comparison to a typical organic liquid electrolyte [227]*.* Copyright 2016, American Chemical Society. **b** Various inorganic solid electrolyte damage mechanisms that can occur as a result of cycling [232]*.* Copyright 2015, American Chemical Society. **c** The electrochemical stability window of some inorganic solid state lithium-ion conductors grouped by the anion type, along with the typically more stable but non-conducting binary compounds [233]*.* Copyright 2019, Springer Nature

A large number of crystalline ceramic electrolytes have been explored, including various structures based on LISICON, NASICON, garnet, perovskite and argyrodite [227–229]. While the room temperature ionic conductivity was initially an issue, conductivities on the order of 10^−3^–10^–2^ S cm^−1^, comparable to liquid organic electrolytes, has been achieved in many of these systems (Fig. 16a). Sulphide-based glass electrolytes are generally able to achieve higher ionic conductivities, although the highest conductivities remain on the order of 10^−3^–10^−2^ S cm^-1^ and their high moisture sensitivity makes processing more challenging [230]. Oxynitride-based glass electrolytes on the other hand have lower conductivities on the order of 10^−6^ S cm^−1^, but are more easily processed into very thin films, such that current capabilities are not compromised at the cell level [231].

The high stiffness of ceramic electrolytes makes them excellent for the suppression of dendritic growth, but also makes intimate electrode contact more challenging, leading to high contact resistance at the electrode-electrolyte interface. The poor contact worsens with successive charge/discharge cycles as the repeated strain cycles on the stiff electrolyte, associated with volume expansion of the electrode, gradually lead to mechanical damage in the form of cracks or delamination from the electrode (Fig. 16b). Non-uniform plating of the metallic anode can also still occur through inhomogeneities such grain boundaries, pores, or even cracks formed during cycling [232]. In comparison, glass electrolytes face less interfacial contact issues due to higher processability and are less susceptible to inhomogeneity issues related to grain boundaries. However, the lower stiffness, while beneficial for alleviating mechanical damage over repeated strain cycles, also makes them less able to stop dendritic growth [230, 231]. General strategies to address the contact issue involve high-pressure processing [234], addition of a more conformable polymer or liquid interface at the electrode [235] or direct combination of the active materials and solid electrolyte within the electrode as an anolyte or catholyte [236–238].

Electrochemical stability is also an issue, with the generally highly complex polyatomic lithium conductors undergoing redox reactions into simpler, more stable compounds at the electrodes. In general, oxide and chloride electrolytes show much larger ESWs than sulphide electrolytes, but remain susceptible to decomposition, with full stability against lithium metal in the 0.2–4.5 V window of typical LIBs being fairly rare (Fig. 16c). The general suppression method through kinetic control is similar to that found in aqueous electrolytes. However, reducing the availability of the reactive species through WiSE-like approaches is not possible. Instead, control is mainly done at the interface, either by reducing the interfacial electronic conductivity through tuning of electrode and electrolyte compositions, or direct deposition of artificial SEIs [234].

Although much progress has been made on the resolution of the aforementioned issues, cycle stability remains an issue for inorganic solid-state LIBs, with examples typically exhibiting cycle lives of 100 or less [229, 231] for both sulphide glass and various oxide ceramic electrolytes. Thin film oxynitride batteries are able to exhibit excellent cyclability of up to 90% retention over 1000 cycles with high voltage LCO cathodes [239]; however, their morphology limits their application to micro-batteries [240].

#### Organic and Composite Solid Electrolytes

**Fig. 17:**
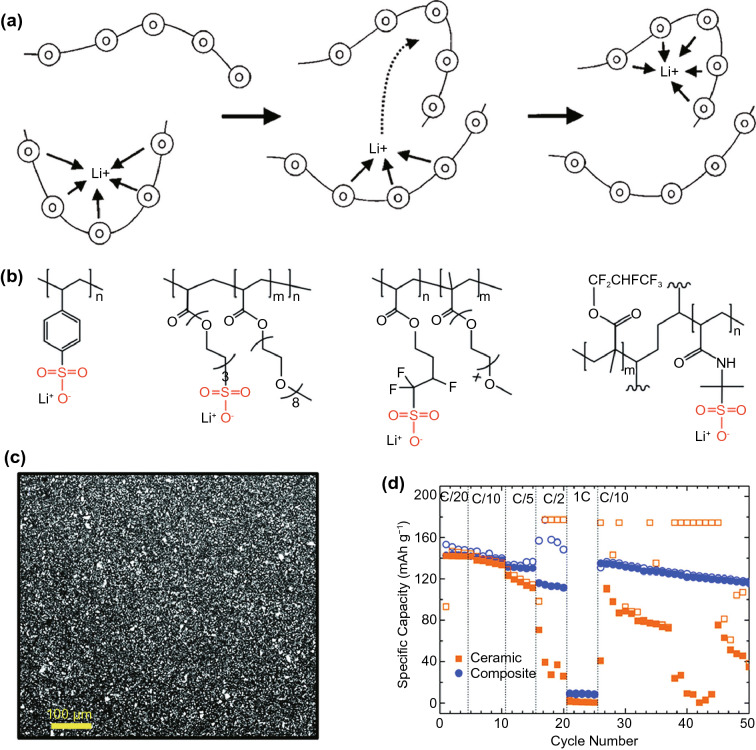
**a** Illustration of the ion conduction mechanism for dilute polymer solid electrolytes [241]*.* Copyright 2016, Elsevier. **b** Schematic of selected sulphonate-based polymer electrolytes. Notice the various side group modifications that affect the charge density of the sulphonate group, thus affecting the coordination strength with the lithium ion [243]*.* Copyright 2018, Royal Society of Chemistry. **c** Scanning electron micrograph of a polymer–ceramic composite electrolyte, with its **d** rate performance exceeding a pure ceramic electrolyte despite having a lower ionic conductivity, due to its ability to be processed into a thinner membrane. Open and filled symbols represent charge and discharge capacities, respectively [238]*.* Copyright 2018, Royal Society of Chemistry

Polymer electrolytes usually consist of an active ion salt that is in solid solution with the polymer, with the ions being coordinated with polar groups on the polymer and their movement being facilitated by the thermal segmental motion of the chains (Fig. 17a) [230]. The involvement of the polymer chain in ionic conduction is markedly different from inorganic solid electrolytes, where the atoms of the glass or ceramic structure do not move during ion diffusion. Appreciable ionic conductivity in polymers is thus usually only observed above the glass transition temperature, where mechanical properties tend to be worse. Compared to inorganic electrolytes, polymer electrolytes are much easier to process, and their mechanical flexibility lends well to maintaining contact and avoiding damage at the interface. Electrochemical stability is also generally fairly high. However, the same flexibility also leads to poorer resistance to dendrite penetration, and a major issue is faced with low ionic conductivities [241, 242]. While generally less flammable than liquid organic electrolytes, polymer electrolytes nonetheless do not fully address the flammability issue.

A large variety of polymers have been explored, with many based on polyethylene oxide, polycarbonates and polysiloxanes [241–243]. Mechanical properties are improved by usual polymeric methods, such as crosslinking, while maintaining sufficient chain mobility for ionic conductivity. Ionic conductivity is improved by increasing chain mobility and tuning the strength of the interaction between the polymer polar groups and the active ion via various chain modifications (Fig. 17b), so that it is strong enough for appreciable ion solubility, and yet not so strong that the ions become trapped. However, despite these efforts, ionic conductivity remains generally in the region of 10^−5^ to 10^−4^ S cm^−1^, substantially lower than inorganic electrolytes, although this may be mitigated at the cell level due to easier processing into thin films [238]. An interesting recent development is the increase of the active ion salt concentration in the electrolyte to more than 50%, forming a polymer-in-salt electrolyte (PiSE). This forms numerous ion clusters within the polymer, instead of individual ions coordinated to polar groups, and allegedly changes the dominant conduction mechanism from segment motion-assisted diffusion to direct hopping of ions from cluster to cluster. Nonetheless, ionic conductivities remain in the region of 10^−4^ S cm^−1^ [244].

A direct method of improving polymer electrolyte conductivities is to composite it with ion-conducting inorganic electrolytes. Compositing methods range from simple mixtures to 3D inorganic frameworks that are then infiltrated with the polymer, and can improve the ionic conductivity of polymer electrolytes by 1–3 orders of magnitude. This combines the high conductivity of inorganic electrolytes with the easy processability of polymer electrolytes, potentially leading to better cell-level performance (Fig. 17c, d). Nonetheless, as with most composites, the properties lie in-between that of the matrix and reinforcement, and final conductivities achieved remain in the region of 10^−4^ S cm^−1^ [245, 246].

In general, while the reported results vary substantially among studies, polymer and composite electrolyte LIBs can achieve slightly better cycle lives than inorganic electrolyte LIBs, with capacity retention exceeding 90% over a few hundred cycles more commonly reported than the latter [229, 242, 246]. This is nonetheless insufficient for the applications that LIBs are currently used for. It should be noted that lithium–polymer (LiPo) batteries, which are fairly common in industry, also encompass typical salt-in-organic solvent electrolytes that are immobilised by a polymer into a gel and are not strictly solid-state, as ion conduction still occurs through the liquid. These do not offer the same safety benefits as true solid-state and aqueous electrolytes due to the high liquid organic content.

## The Long Road to Commercialisation

**Fig. 18:**
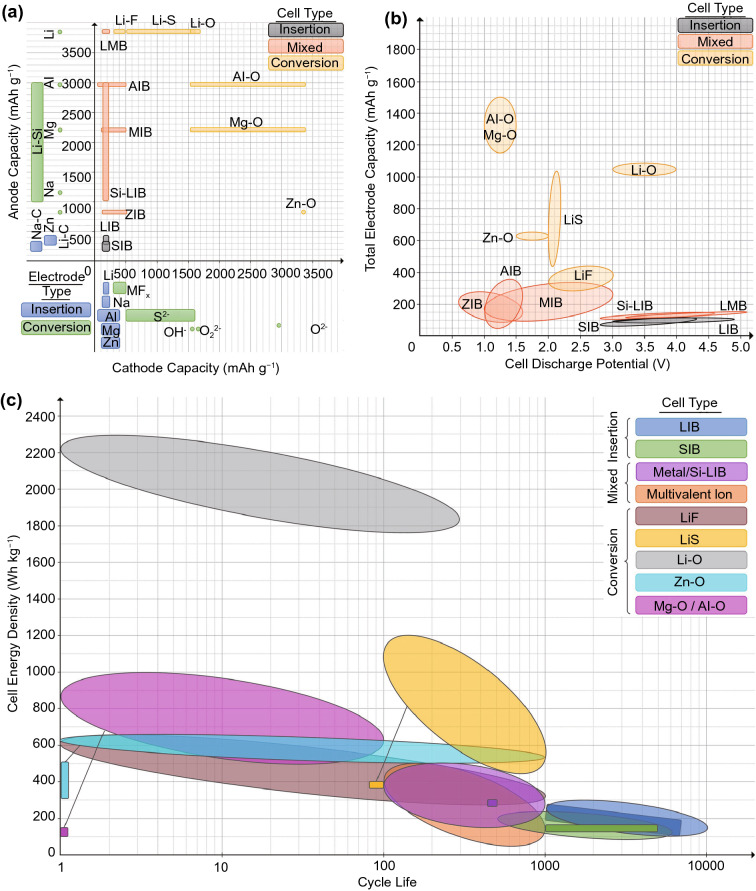
General maps showing **a** charge capacities of various anodes and cathodes along with promising battery chemistries that can be formed from their combination. The charge capacities of metallic anodes and oxygen cathodes are their theoretical values, while all other electrodes are reported values in academia as covered in this review. For consistency, all active ion masses for insertion electrodes are assigned to the anode. **b** Total electrode capacities calculated from **a** for the aforementioned combinations, plotted against reported discharge voltages as covered in this review. **c** Cell energy densities calculated from **b** assuming 50% inactive mass, plotted against reported cycle lives as covered in this review. The faded oval regions represent the general performance of the cell chemistry as reported in academia, while solid rectangular regions represent the performance of commercially available cells [36, 37, 210, 247–256]*,* illustrating the gaps between them

LIBs currently enjoy far better all-around performance than any contemporary alternatives such as lead-acid and nickel metal hydride batteries, allowing them to become the workhorse for various mobile, portable and wearable devices over the past few decades. This dominance is likely to continue well into the coming decade, especially with increasingly demanding applications arising from electrification of the transport and energy industries. Nonetheless, issues remain with cost, capacity and safety, and there is an apparent need to explore “beyond lithium-ion” alternatives that are far better than what is currently available. Given the essential components for a battery are the electrodes and electrolytes, understanding their fundamental chemistry, along with management of their interfaces, are key parts of the systematic approach that has been adopted through this review. The position of elements on the periodic table is a useful indicator of their theoretical energy densities, and can be used to identify suitable alternative ions for insertion batteries or conversion electrode materials, while simultaneously considering their cost. After selection of the electrodes, suitable electrolytes with suitable ionic conductivities, ESWs and other physical properties such as flammability can be determined. This bottom-up process of cell design from electrode pairing to final cell performance is summarised in Fig. 18, showing the general regions of performance that have currently been achieved for the promising combinations discussed in Sects. 3–5, as well as a comparison to emerging commercial examples of such cells.

Several general conclusions are apparent from Fig. 18. The pure insertion electrode batteries, LIBs and SIBs, suffer from low charge capacities of both the anode and cathode, although this is compensated for by high discharge voltages. Substituting the graphitic anode with a high capacity conversion anode such as lithium metal or silicon only produces a small change in the total charge capacity due to the low capacity cathode. The multivalent-ion batteries, AIBs, MIBs and ZIBs, which are also mixed batteries with metallic conversion anodes and insertion cathodes, suffer similarly from low total capacities. Interestingly, the capacities are only weakly correlated with the charge capacity of the active ion, implying that the insertion frameworks remain a substantial proportion of the total electrode mass. Very high total charge capacities are only achieved where both the anode and cathode are conversion electrodes. However, these suffer when it comes to cyclability (Fig. 18c), where pure insertion electrode batteries show by far the best rechargeability, being regularly able to achieve cycle lives of several thousand or more. Notably, the introduction of just one conversion electrode into the cell design immediately reduces the cycle life to the order of 10^2^ or less. The relative maturity of these promising chemistries can also be seen by comparing the performances achieved in laboratories and what is available commercially (Fig. 18c). Commercial and laboratory LIB performances essentially overlap, illustrating their maturity but also showing the limits of the technology and the need for better alternatives.

### Progress with Alternative Ions

Among all the possible alternative active ions for insertion battery, SIBs are the only ones that are currently commercially available, although the market volume is rather small. Aqueous SIBs were the first to be commercialised, but did not gain substantial attention due to the low achievable energy densities. Current SIBs are close analogues to conventional LIBs, with disordered soft or hard carbon anodes and typically layered oxide cathodes in an organic liquid electrolyte [249, 257]. Nearly all performance parameters are able to match LIBs, with cycle lives of several thousand and discharge rates of up to 5C. Operating temperatures and self-discharge rates are also similar. However, the energy densities achieved of about 150 Wh kg^−1^ [249, 250] is only about 60% of layered oxide LIBs. This is within expectations from academic results (Fig. 18c), and appears to be a consequence of lower discharge voltages rather than lower electrode charge capacities that might be expected to arise from sodium’s larger ionic radius (Fig. 18b). Nonetheless, commercial SIBs can exhibit a much larger discharge window than LIBs, enabled by the use of an aluminium instead of the less stable copper current collector [257], which may alleviate issues with over-discharge that LIBs sometimes face.

Remarkably, the total electrode charge capacities achievable by multivalent-ion batteries appear to be capable of exceeding even LIBs with high capacity metal and silicon anodes (Fig. 18b), as a result of a higher limit for cathode capacities (Fig. 18a). However, like sodium, the overall energy densities suffer from low discharge voltages. Commercially, zinc is the most mature, with start-up companies searching for commercially viable ZIBs that nonetheless remain elusive, likely due to low achievable energy densities at the cell level. Magnesium and aluminium continue to be plagued by unresolved electrolyte issues and the lack of simultaneously high capacity and high cycle life cathodes. While Grignard-based and ionic liquid electrolytes work in the academic environment, their high cost and corrosive nature make commercialisation difficult. The vast majority of cathodes that have been explored are also not synthesised with the active ion as part of the structure, unlike in LIBs and SIBs. This necessitates the use of metallic anodes as the ion source in the cell, which, while attractive from the energy density point of view, adds more unresolved issues to the system.

A significant obstacle to the progress of these alternative ion batteries could well be the lack of a systematic method for the discovery of new insertion electrodes, as opposed to the periodic table approach that can be taken for conversion electrodes, with current cathode designs typically taking inspiration from successful examples in LIBs. New approaches may be considered, such as the combination of conventional molecular dynamics and density functional theory simulations with emerging machine and deep learning techniques, which are well-suited to tackle such complex and highly multivariate problems. Such methods may aid the discovery of new high-energy, high cycle life cathodes that improve the energy densities of alternative ion batteries and accelerate their commercialisation process.

**Fig. 19:**
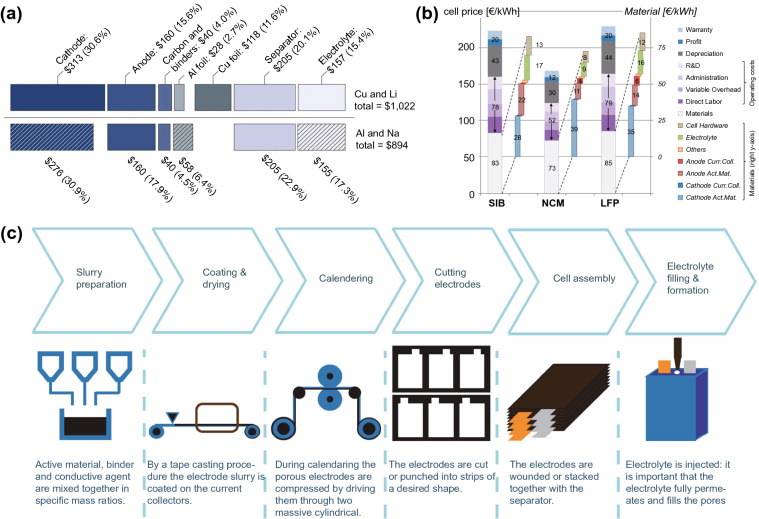
**a** Cost comparison of a hypothetical SIB formed from simple replacement of Li and Cu to Na and Al in an LIB [42]*.* Copyright 2018, Springer Nature. **b** Cost comparison of an SIB with NCM and LFP LIBs [259]*.* Creative Commons License (CC BY 4.0). **c** Typical manufacturing process for a commercial LIB [260]*.* Creative Commons License (CC BY 4.0)

At the moment, the cost advantage of these alternative ion batteries is also unclear, as while SIBs are commercially available, they do not yet enjoy the same economies of scale as LIBs. The BatPaC model [258] calculates cell prices from raw material costs with some informed assumptions on production costs, which if kept the same for LIBs and SIBs, can be used to sidestep the issue of scale. It has been found using a modified version of this model that simple substitution of the lithium and copper in an LIB for sodium and aluminium while keeping all other costs identical, only results in an approximately 12% reduction in price (Fig. 19a). This shows that the massive price difference between metallic lithium and sodium in fact carries over fairly poorly to cell prices, simply because of the significant cost of other components and manufacturing processes [42]. Considering more realistic SIB chemistries shows that their prices per unit energy can actually be higher than NCM LIBs due to lower capacities (Fig. 19b) [259]. The situation is analogous in other multivalent ion systems, illustrating the need for higher capacity electrodes and to consider the cost of all components of the cell, rather than just the use of a cheaper active ion, if the goal of a cheaper LIB alternative is to be achieved.

In addition, while alternative ion batteries continue to improve, LIB development is not stagnant. The continuous reduction in LIB prices as manufacturers strive towards the Energy Storage Grand Challenge targets presents a shifting goalpost for the alternative ions. Interest in recycling [261] and new extraction techniques is also growing [262, 263] and may prove to be a more direct answer to lithium resource depletion than new battery chemistries.

### Progress with Conversion Anodes

While battery manufacturers have begun adding small amounts of silicon to conventional graphite anodes to form composite anodes in otherwise conventional LIBs, pure silicon anodes of the sort commonly reported in literature remain relatively rare. Among these, only one example with published performance figures, by Enovix [247], is known, achieving cell-level capacities of approximately 300 Wh kg^−1^ with a cycle life of 500–80% initial capacity at 25 °C, and a continuous discharge capability of slightly less than 1C. A fairly commonly achieved anode capacity in academia of 2000 mAh g^−1^, about 540% that of conventional graphite, would result in an approximately 20% increase in cell energy density, in the case of the anode active mass accounting for 20% of the total cell mass [264, 265], and all other cell components remaining the same. Hence, the 300 Wh kg^−1^ capacity achieved is well in line with expectations assuming the other cell components being largely similar to typical 250 Wh kg^−1^ [252] graphite anode LIBs. The cycle life, like the energy density, is generally in good agreement with expectations from academic results (Fig. 18c). However, it is unclear if relatively the small increase in capacity will be considered worth the fairly large trade-off in cycle life and current capabilities to the industry in general. This is especially since conventional cylindrical LIBs can currently achieve energy densities of as high as 280 Wh kg^−1^ under deep discharge, although current capabilities and cyclability at such high capacities are low [36]. Hence, pairing with higher charge capacity or higher voltage cathodes may be necessary to further increase the attractiveness of silicon anodes. Price is also a concern, as the continuous, high-throughput tape-casting and calendaring process used for typical LIB electrodes (Fig. 19c) is substantially more difficult to implement with the nanostructured electrodes that are so common in academia, and 0D nanoparticulate silicon anodes are likely to face smaller barriers to commercialisation.

### Progress with Solid and Aqueous Electrolytes

The commercial development of solid electrolytes has proceeded hand-in-hand with metallic anodes, as the former can enable the use of the latter. A fairly large number of companies have entered the space, including both established battery manufacturers and start-ups. These companies typically choose to use lithium metal anodes together with solid electrolytes and conventional LIB insertion cathodes, forming an all-solid-state battery (ASSB) with the promise of improving both safety and capacity. The majority of these are based on inorganic electrolytes, with sulphides being generally more popular than oxides, and with each company having their proprietary, undisclosed formulations [266]. Unfortunately, despite the large amount of interest, published performance figures are extremely scarce and a set with both capacity and cycle life remains unavailable, making comparisons with contemporary LIBs challenging. Polymer ASSBs, while less popular, have slightly longer histories, with Blue Solutions having produced an example for more than a decade [251]. However, while cell-level performance data are not available, all performance parameters at the pack level, including capacity of ~140 Wh kg^−1^, cycle life of 4000 and a discharge rate of ~1 C, appear highly similar to contemporary LIBs [37, 253–255] and generally not in line with expectations from academic results. The reason for this cannot be deduced without further information on the electrolyte chemistry and battery architecture. Nonetheless, it should be noted that like silicon anodes, the energy density of lithium metal batteries is limited to an approximately 30% improvement over LIBs if there are no significant changes in the cathode or packaging, arising from a 20% reduction in cell weight due to the removal of the graphite anode and a slightly higher cell potential. Hence, if a lithium metal ASSB is designed with low electrode mass loading or thick electrolyte layers, the theoretical capacity advantage can easily be eroded.

Aqueous LIBs have received far less recent attention than ASSBs, and commercial cells are rare. A notable example is the LTO anode aqueous LIB under development at Toshiba [267], utilising a WiS-esque concentrated salt electrolyte. The usage of an LTO anode places it in a different application category from graphite anode LIBs, and its less negative reduction potential conveniently sidesteps the stability issues at the anode that WiSEs generally face. The cycle life is impressive, showing near 100% capacity retention after 2000 cycles, although the cathode and cell-level energy density has not been disclosed.

### Progress with Conversion Cathodes

Batteries using conversion cathodes usually also use conversion anodes, leading to substantially higher charge capacities and energy densities than the insertion and mixed electrode batteries previously covered (Fig. 18c). Among these, lithium–sulphur batteries stand out as a chemistry that has achieved the best balance of energy density and cycle life in academia so far. The performance of the metal–air batteries depends heavily on the anode, with lithium–air batteries achieving extremely high energy densities due to the high cell discharge voltage, but relatively poor cycle life. Zinc–air batteries, on the other hand, generally exhibit better rechargeability but lower energy density due to the lower energy zinc anode. Aluminium and magnesium–air batteries, being generally newer technologies than lithium and zinc–air, do not compare favourably, with slightly higher energy densities than zinc–air but far poorer cyclability. Lithium metal halide batteries, likewise, do not compare favourably in both energy density and cycle life to the more successful zinc–air and lithium–sulphur chemistries.

Commercially, examples of these high energy conversion cathode batteries remain rare. Among the solid conversion cathodes, only lithium–sulphur has established any commercial presence, and lithium metal halide batteries remain unavailable. Battery manufacturer Oxis is notable as one of the first to publish a complete set of cell-level performance figures for lithium–sulphur batteries. Its cell is also particularly interesting as it employs several advanced techniques explored in academia to a commercial cell, including artificial SEIs for the lithium metal anode and nanostructured sulphur at the cathode [175]. The achieved cell capacities are as high as 400 Wh kg^−1^, considerably higher than both LIBs and current silicon anode batteries, with continuous discharge rates of 1C, although the cycle life of less than 100 makes it unsuitable for replacing LIBs [248]. Notably, the energy density and cycle life performance are substantially worse than what is generally observed in academia. The lower energy density indicates that the amount of inactive mass in the cell is far higher than the approximately 50% that is typical of LIBs [264, 265] and which is assumed in Fig. 18c. This starkly illustrates the importance of achieving high electrode mass loading and minimising electrolyte use while designing strategies to improve sulphur utilisation and cycle life. The lower cycle life also indicates potential issues with the rapid cycling tests that are commonly done in academia, which may not reflect charge-discharge conditions in real applications, indicating the need for long-term cycling investigations in addition to the typical rapid tests. This appears to be a larger issue for lithium–sulphur batteries than other chemistries, likely due to kinetic nature of the polysulphide shuttling problem. Finally, nanostructured sulphur cathodes are likely to face the same issues with commercialisation as silicon anodes due to challenges with high-throughput production processes. Hence, like silicon anodes, nanoparticulate cathodes should not be completely neglected despite their tendency for worse performance than nanostructured cathodes.

Rechargeable metal–air batteries, facing difficult simultaneous issues with the metallic anode, electrolyte and air cathode, have not yet established any commercial presence, although numerous start-up attempts have been made. The popular method of stabilising metallic anodes with a solid electrolyte is also much more difficult to implement with an air cathode than with solid cathodes. While rechargeable zinc–air [268] and iron–air [269] batteries are being actively explored for grid energy storage, commercial examples for high-energy applications are not known. It should be noted that the key performance factor for batteries for grid storage is their round-trip efficiency, for which air cathodes perform generally quite poorly at in academia.

Existing primary zinc–air batteries are able to achieve energy densities of up to approximately 400 Wh kg^−1^ [210, 256], which, while higher than LIBs, is lower than primary lithium metal batteries at approximately 600 Wh kg [270], making them less attractive for high-energy applications. High-capacity aqueous primary batteries, utilising higher energy metal anodes such as magnesium and aluminium instead of zinc, have thus also been a popular development. The design goal for these is usually for the ability to recharge via mechanical replacement of the anode. Hence, while unable to replace LIBs, these may find use in niche applications requiring very high energy capacity with promised energy densities exceeding 1000 Wh kg^−1^ in the case of aluminium [271]. However, published cell-level performance figures remain generally unavailable, including cycle stability of the cathode, which is important if mechanical recharging of the anode is to be accomplished. Aqueous magnesium–air batteries have been commercialised as emergency batteries, but are only able to achieve capacities in the region of 100–150 Wh kg^−1^ [272–274]. Like lithium–sulphur, the energy density of these primary commercially available metal–air batteries is far worse than what is expected from laboratory results, again illustrating the impact that inactive battery mass, which is often omitted in academia, can have on the overall energy density of the cell.

The commercial success of metal–air batteries will also be dependent on their current capabilities. A typical 18650 cylindrical LIB with a total electrode area of approximately of 500 cm^2^ [275, 276] and an energy capacity of 11.4 kW would require an electrode power density of approximately 23 mW cm^−2^ to discharge at 1 C, a reasonable peak discharge rate for most applications. An air battery of the same geometry would have a much lower cathode area of around 40 cm^2^ due to the inability to coil the cathode, corresponding to the external surface area of the 18650 cell, requiring a power density of close to 300 mW cm^−2^ to deliver the same power as a typical LIB at 1 C. This, at first glance, is not far from the values that are being achieved in academia under galvanodynamic testing. However, it should be noted that commercial zinc–air batteries of energy densities exceeding 300 Wh kg^−1^, utilising MnO_2_ catalysts that can exhibit around 100 mW cm^−2^ under similar testing conditions, are only able to achieve continuous discharge rates of less than 0.05 C [210, 256]. Much of this discrepancy is due to instantaneous oxygen availability, and pulse currents can be close to 0.5 C [210]. This is more in line with expectations from galvanodynamic measurements, and indicates that the power limitations faced by commercial air-cathode batteries are due at least as much to insufficient rates of oxygen supply as low ORR catalyst activity. Resolving this, along with other practical issues like electrolyte contamination, may require engineering solutions such as forced induction or air filters, compromising the energy density of the energy storage system as a whole.

## Summary and Perspectives

Through a systematic approach, suitable materials and elements for high-energy “beyond lithium-ion” batteries have been identified and correlated with cell-level developments in academia and industry, each of which have their advantages and limitations compared with LIBs as the benchmark. There are alternative ions to lithium that promise lower costs. However, the capacities of SIBs remain substantially lower than LIBs. While the theoretical understandings of the topics have improved considerably, the multifaceted challenges faced by multivalent-ion batteries such as ZIBs, MIBs and AIBs in the electrodes and electrolyte have so far been insurmountable as a whole. New, computer-driven methods of searching for solutions may be useful in speeding up this process. Solely replacement of the active ion may also not produce significant cost savings, and care must be taken that low-cost electrolytes and electrodes are also utilised.

Conversion electrodes are expected to lead to significantly higher cell capacities, and the commonly faced issues of low conductivity, electrode disintegration and dissolution are slowly being successfully resolved. Silicon and metal anode LIBs have begun appearing on the market. However, capacity improvements appear to be rather modest in both cases, even though commercial silicon anode batteries appear to be performing approximately as expected from academic results. This appears to be due to the upper limit of these improvements being removal of the graphite anode weight in LIBs, which is not a sufficiently large proportion of the overall cell weight, and simultaneous usage of higher capacity conversion cathodes is necessary for larger improvements. The conversion cathodes, however, suffer from limited rechargeability, with the cycle life of sulphur cathodes, for example, being limited to 100 commercially for the time being. Higher cycle lives observed in academia could be due to the shorter time scales at which they are tested as compared to commercial applications. A rift is also seen in capacities achieved in academia and industry, suggesting the former may be underestimating the amount of inactive mass required in the solutions designed. Halide cathodes suffer from greater issues and see substantially less success in both academia and industry. The challenges facing rechargeable oxygen cathodes in the form of metal–air batteries have likewise proven to be too difficult for commercialisation. Primary metal–air batteries continue to be plagued by very low power density, and this appears to be due to engineering challenges related to air supply, on top of the intrinsic reaction rate limitations of the cells and catalysts.

Alternative electrolytes promise better safety and may also address several issues with conversion electrodes. Expansion of the ESW for aqueous electrolytes remains an issue, and current commercial examples operate at lower voltages than typical LIBs with organic electrolytes. Solid electrolytes have seen rapid development and have played a major role in enabling metal anode batteries commercially. Although cell-level performance parameters are currently unavailable for a full comparison to LIBs, these are likely to become available within the next few years.

When it comes to the overall combination of energy, power, cyclability and cost, there is little doubt that LIBs remain the gold standard for the time being. Nonetheless, progress in many of these “beyond lithium-ion” alternatives has been encouraging, although clear gaps to commercialisation remain in each case. As these remaining issues continue to be resolved for the wider spectrum of end-users that are emerging in the exponentially growing battery market, several of these frontrunners could very well coexist alongside LIBs or even begin to challenge their dominance, as early as within the decade.

## Footnotes

1 The term “energy density” presently refers to gravimetric energy density, unless otherwise stated.

2 The term “charge capacity” presently refers to gravimetric charge density, unless otherwise stated.

## References

[1] A. Volta, On the electricity excited by the mere contact of conducting substances of different kinds. In a letter from Mr. Alexander Volta, F. R. S. Professor of Natural Philosophy in the University of Pavia, to the Rt. Hon. Sir Joseph Banks, Bart. K. B. P. R. S. Proc. Royal Soc. Lond. **1**, 27–29 (1832). 10.1098/rspl.1800.0016

[2] Zhao Y, Pohl O, Bhatt AI, Collis GE, Mahon PJ (2021). A review on battery market trends, second-life reuse, and recycling. Sustain. Chem..

[3] Shanghai metals market (Shanghai Metals Market, 2021). https://www.metal.com. Accessed 24 July 2021

[4] Preismonitor Mai 2021 (Deutsche Rohstoffagentur, 2021). https://www.deutsche-rohstoffagentur.de/DERA/DE/Produkte/Rohstoffpreise/Preismonitor/preismonitor_node.html. Accessed 25 July 2021

[5] Mineral commodity summaries 2021 (U.S. Geological Survey, 2021). https://pubs.er.usgs.gov/publication/mcs2021. Accessed 25 July 2021

[6] Average prices over the last few months for rare earth. (Institut für seltene Erden und Metalle AG, 2021). https://ise-metal-quotes.com. Accessed 26 July 2021

[7] The future of hydrogen. (International Energy Agency, 2019). https://www.iea.org/reports/the-future-of-hydrogen. Accessed 25 July 2021

[8] China: market price: monthly avg: inorganic chemical material: yellow phosphorus, 99.9% or above. (CEIC Data, 2021). https://www.ceicdata.com/en/china/china-petroleum--chemical-industry-association-petrochemical-price-inorganic-chemical-material/cn-market-price-monthly-avg-inorganic-chemical-material-yellow-phosphorus-999-or-above. Accessed 25 July 2021

[9] Hwang JY, Myung ST, Sun YK (2017). Sodium-ion batteries: present and future. Chem. Soc. Rev..

[10] Saha P, Datta MK, Velikokhatnyi OI, Manivannan A, Alman D (2014). Rechargeable magnesium battery: current status and key challenges for the future. Prog. Mater. Sci..

[11] Yang H, Li H, Li J, Sun Z, He K (2019). The rechargeable aluminum battery: opportunities and challenges. Angew. Chem. Int. Ed..

[12] Winsberg J, Hagemann T, Janoschka T, Hager MD, Schubert US (2017). Redox-flow batteries: from metals to organic redox-active materials. Angew. Chem. Int. Ed..

[13] Nowroozi MA, Mohammad I, Molaiyan P, Wissel K, Munnangi AR (2021). Fluoride ion batteries—past, present, and future. J. Mater. Chem. A.

[14] Amatucci GG, Pereira N (2007). Fluoride based electrode materials for advanced energy storage devices. J. Fluor. Chem..

[15] Rahman MA, Wang X, Wen C (2013). High energy density metal-air batteries: a review. J. Electrochem. Soc..

[16] Manthiram A (2020). A reflection on lithium-ion battery cathode chemistry. Nat. Commun..

[17] Chung SH, Manthiram A (2019). Current status and future prospects of metal–sulfur batteries. Adv. Mater..

[18] Balogun MS, Qiu W, Wang W, Fang P, Lu X (2014). Recent advances in metal nitrides as high-performance electrode materials for energy storage devices. J. Mater. Chem. A.

[19] Fu Y, Wei Q, Zhang G, Sun S (2018). Advanced phosphorus-based materials for lithium/sodium-ion batteries: recent developments and future perspectives. Adv. Energy Mater..

[20] Brandt K (1994). Historical development of secondary lithium batteries. Solid State Ion..

[21] Schweidler S, Biasi L, Schiele A, Hartmann P, Brezesinski T (2018). Volume changes of graphite anodes revisited: a combined operando X-ray diffraction and in situ pressure analysis study. J. Phys. Chem. C.

[22] Asenbauer J, Eisenmann T, Kuenzel M, Kazzazi A, Chen Z (2020). The success story of graphite as a lithium-ion anode material—fundamentals, remaining challenges, and recent developments including silicon (oxide) composites. Sustain. Energy Fuels.

[23] Mao C, Ruther RE, Li J, Du Z, Belharouak I (2018). Identifying the limiting electrode in lithium ion batteries for extreme fast charging. Electrochem. Commun..

[24] Ohzuku T, Ueda A, Yamamoto N (1995). Zero-strain insertion material of Li[Li_1/3_Ti_5/3_]O_4_ for rechargeable lithium cells. J. Electrochem. Soc..

[25] Nitta N, Wu F, Lee JT, Yushin G (2015). Li-ion battery materials: present and future. Mater. Today.

[26] Armstrong AR, Bruce PG (1996). Synthesis of layered LiMnO_2_ as an electrode for rechargeable lithium batteries. Nature.

[27] Dutta G, Manthiram A, Goodenough JB, Grenier JC (1992). Chemical synthesis and properties of Li_1−δ−x_Ni_1+δ_O_2_ and Li[Ni_2_]O_4_. J. Solid State Chem..

[28] Purwanto A, Yudha CS, Ubaidillah U, Widiyandari H, Ogi T (2018). NCA cathode material: synthesis methods and performance enhancement efforts. Mater. Res. Express.

[29] Li W, Liu X, Celio H, Smith P, Dolocan A (2018). Mn versus Al in layered oxide cathodes in lithium-ion batteries: a comprehensive evaluation on long-term cyclability. Adv. Energy Mater..

[30] Saldaña G, Martín JIS, Zamora I, Asensio FJ, Oñederra O (2019). Analysis of the current electric battery models for electric vehicle simulation. Energies.

[31] Benedek R, Thackeray MM (2006). Reaction energy for LiMn_2_O_4_ spinel dissolution in acid. Electrochem. Solid State Lett..

[32] Doughty DH, Roth EP (2012). A general discussion of Li ion battery safety. Electrochem. Soc. Interface.

[33] Preger Y, Barkholtz HM, Fresquez A, Campbell DL, Juba BW (2020). Degradation of commercial lithium-ion cells as a function of chemistry and cycling conditions. J. Electrochem. Soc..

[34] Islam MS, Fisher CAJ (2013). Lithium and sodium battery cathode materials: computational insights into voltage, diffusion and nanostructural properties. Chem. Soc. Rev..

[35] Lain MJ, Brandon J, Kendrick E (2019). Design strategies for high power vs. high energy lithium ion cells. Batteries.

[36] R. Bugga, C. Krause, K. Billings, J.P. Ruiz, E. Brandon et al., Performance of commercial high energy and high power Li-ion cells in jovian missions encountering high radiation environments. NASA Battery Workshop, Huntsville, Alabama (2019). https://www.nasa.gov/sites/default/files/atoms/files/3-nasa_battery_workshop_nov_2019_high_power_li-ion_cells_final.pdf

[37] ESS batteries by Samsung SDI (Samsung SDI, 2019). https://www.samsungsdi.com/ess/energy-storage-system-reference.html. Accessed 25 July 2021

[38] Hall F, Touzri J, Wußler S, Buqa H, Bessler WG (2018). Experimental investigation of the thermal and cycling behavior of a lithium titanate-based lithium-ion pouch cell. J. Energy Storage.

[39] Haidl P, Buchroithner A, Schweighofer B, Bader M, Wegleiter H (2019). Lifetime analysis of energy storage systems for sustainable transportation. Sustainability.

[40] Ziegler MS, Trancik JE (2021). Re-examining rates of lithium-ion battery technology improvement and cost decline. Energy Environ. Sci..

[41] Energy storage grand challenge (U.S. Department of Energy, 2020). https://www.energy.gov/energy-storage-grand-challenge/articles/energy-storage-grand-challenge-roadmap. Accessed 25 July 2021

[42] Vaalma C, Buchholz D, Weil M, Passerini S (2018). A cost and resource analysis of sodium-ion batteries. Nat. Rev. Mater..

[43] Rechargeable batteries (Cobalt Institute, 2021). https://www.cobaltinstitute.org/rechargeable-batteries.html. Accessed 25 July 2021

[44] Depcik C, Cassady T, Collicott B, Burugupally SP, Li X (2020). Comparison of lithium ion Batteries, hydrogen fueled combustion engines, and a hydrogen fuel cell in powering a small unmanned aerial vehicle. Energy Convers. Manag..

[45] Galushkin NE, Yazvinskaya NN, Galushkin DN (2018). Mechanism of thermal runaway in lithium-ion cells. J. Electrochem. Soc..

[46] Feng X, Ouyang M, Liu X, Lu L, Xia Y (2018). Thermal runaway mechanism of lithium ion battery for electric vehicles: a review. Energy Storage Mater..

[47] Zhang W, Liu Y, Guo Z (2019). Approaching high-performance potassium-ion batteries via advanced design strategies and engineering. Sci. Adv..

[48] Ji B, He H, Yao W, Tang Y (2021). Recent advances and perspectives on calcium-ion storage: key materials and devices. Adv. Mater..

[49] Che H, Chen S, Xie Y, Wang H, Amine K (2017). Electrolyte design strategies and research progress for room-temperature sodium-ion batteries. Energy Environ. Sci..

[50] Perveen T, Siddiq M, Shahzad N, Ihsan R, Ahmad A (2020). Prospects in anode materials for sodium ion batteries—a review. Renew. Sustain. Energy Rev..

[51] Zhang W, Zhang F, Ming F, Alshareef HN (2019). Sodium-ion battery anodes: status and future trends. EnergyChem.

[52] Doeff MM, Ma Y, Visco SJ, Jonghe LCD (1993). Electrochemical insertion of sodium into carbon. J. Electrochem. Soc..

[53] Thomas P, Ghanbaja J, Billaud D (1999). Electrochemical insertion of sodium in pitch-based carbon fibres in comparison with graphite in NaClO_4_–ethylene carbonate electrolyte. Electrochim. Acta.

[54] Wen Y, He K, Zhu Y, Han F, Xu Y (2014). Expanded graphite as superior anode for sodium-ion batteries. Nat. Commun..

[55] Jache B, Adelhelm P (2014). Use of graphite as a highly reversible electrode with superior cycle life for sodium-ion batteries by making use of co-intercalation phenomena. Angew. Chem. Int. Ed..

[56] Stevens DA, Dahn JR (2001). The mechanisms of lithium and sodium insertion in carbon materials. J. Electrochem. Soc..

[57] Li Z, Bommier C, Chong ZS, Jian Z, Surta TW (2017). Mechanism of Na-ion storage in hard carbon anodes revealed by heteroatom doping. Adv. Energy Mater..

[58] Song J, Wang K, Zheng J, Engelhard MH, Xiao B (2020). Controlling surface phase transition and chemical reactivity of O_3_-layered metal oxide cathodes for high-performance Na-ion batteries. ACS Energy Lett..

[59] Abraham KM (2020). How comparable are sodium-ion batteries to lithium-ion counterparts?. ACS Energy Lett..

[60] Lyu Y, Liu Y, Yu ZE, Su N, Liu Y (2019). Recent advances in high energy-density cathode materials for sodium-ion batteries. Sustain. Mater. Technol..

[61] Xiao J, Li X, Tang K, Wang D, Long M (2021). Recent progress of emerging cathode materials for sodium ion batteries. Mater. Chem. Front..

[62] Xu J, Lin F, Doeff MM, Tong W (2017). A review of Ni-based layered oxides for rechargeable Li-ion batteries. J. Mater. Chem. A.

[63] Tang W, Song X, Du Y, Peng C, Lin M (2016). High-performance NaFePO_4_ formed by aqueous ion-exchange and its mechanism for advanced sodium ion batteries. J. Mater. Chem. A.

[64] Lu J, Chung SC, Nishimura S, Yamada A (2013). Phase diagram of olivine NaxFePO_4_ (0<x<1). Chem. Mater..

[65] Zhu Y, Xu Y, Liu Y, Luo C, Wang C (2012). Comparison of electrochemical performances of olivine NaFePO_4_ in sodium-ion batteries and olivine LiFePO_4_ in lithium-ion batteries. Nanoscale.

[66] Qian J, Wu C, Cao Y, Ma Z, Huang Y (2018). Prussian blue cathode materials for sodium-ion batteries and other ion batteries. Adv. Energy Mater..

[67] Lee HW, Wang RY, Pasta M, Lee SW, Liu N (2014). Manganese hexacyanomanganate open framework as a high-capacity positive electrode material for sodium-ion batteries. Nat. Commun..

[68] Liu Q, Hu Z, Chen M, Zou C, Jin H (2020). The cathode choice for commercialization of sodium-ion batteries: layered transition metal oxides versus Prussian blue analogs. Adv. Funct. Mater.

[69] Ong SP, Chevrier VL, Hautier G, Jain A, Moore C (2011). Voltage, stability and diffusion barrier differences between sodium-ion and lithium-ion intercalation materials. Energy Environ. Sci..

[70] Lahan H, Boruah R, Hazarika A, Das SK (2017). Anatase TiO_2_ as an anode material for rechargeable aqueous aluminum-ion batteries: remarkable graphene induced aluminum ion storage phenomenon. J. Phys. Chem. C.

[71] Kazazi M, Abdollahi P, Mirzaei-Moghadam M (2017). High surface area TiO_2_ nanospheres as a high-rate anode material for aqueous aluminium-ion batteries. Solid State Ion..

[72] Yuan D, Zhao J, Manalastas W, Kumar S, Srinivasan M (2020). Emerging rechargeable aqueous aluminum ion battery: status, challenges, and outlooks. Nano Mater. Sci..

[73] Zhao Q, Zachman MJ, Sadat WIA, Zheng J, Kourkoutis LF (2018). Solid electrolyte interphases for high-energy aqueous aluminum electrochemical cells. Sci. Adv..

[74] Zhang Y, Liu S, Ji Y, Ma J, Yu H (2018). Emerging nonaqueous aluminum-ion batteries: challenges, status, and perspectives. Adv. Mater..

[75] Xing W, Du D, Cai T, Li X, Zhou J (2018). Carbon-encapsulated CoSe nanoparticles derived from metal-organic frameworks as advanced cathode material for Al-ion battery. J. Power Sources.

[76] Jiang J, Li H, Huang J, Li K, Zeng J (2017). Investigation of the reversible intercalation/deintercalation of Al into the novel Li_3_VO_4_@C microsphere composite cathode material for aluminum-ion batteries. ACS Appl. Mater. Interfaces.

[77] Lu H, Wan Y, Wang T, Jin R, Ding P (2019). A high performance SnO_2_/C nanocomposite cathode for aluminum-ion batteries. J. Mater. Chem. A.

[78] Wu C, Gu S, Zhang Q, Bai Y, Li M (2019). Electrochemically activated spinel manganese oxide for rechargeable aqueous aluminum battery. Nat. Commun..

[79] Xing L, Owusu KA, Liu X, Meng J, Wang K (2021). Insights into the storage mechanism of VS_4_ nanowire clusters in aluminum-ion battery. Nano Energy.

[80] Yan C, Lv C, Wang L, Cui W, Zhang L (2020). Architecting a stable high-energy aqueous Al-ion battery. J. Am. Chem. Soc..

[81] Li H, Yang H, Sun Z, Shi Y, Cheng HM (2019). A highly reversible Co_3_S_4_ microsphere cathode material for aluminum-ion batteries. Nano Energy.

[82] Lin MC, Gong M, Lu B, Wu Y, Wang DY (2015). An ultrafast rechargeable aluminium-ion battery. Nature.

[83] Wu F, Yang H, Bai Y, Wu C (2019). Paving the path toward reliable cathode materials for aluminum-ion batteries. Adv. Mater..

[84] Balland V, Mateos M, Singh A, Harris KD, Laberty-Robert C (2021). The role of Al^3+^-based aqueous electrolytes in the charge storage mechanism of MnO_x_ cathodes. Small.

[85] Kong Y, Tang C, Huang X, Nanjundan AK, Zou J (2021). Thermal reductive perforation of graphene cathode for high-performance aluminum-ion batteries. Adv. Funct. Mater.

[86] Shen X, Sun T, Yang L, Krasnoslobodtsev A, Sabirianov R (2021). Ultra-fast charging in aluminum-ion batteries: electric double layers on active anode. Nat. Commun..

[87] Faegh E, Ng B, Hayman D, Mustain WE (2021). Practical assessment of the performance of aluminium battery technologies. Nat. Energy.

[88] Li D, Yuan Y, Liu J, Fichtner M, Pan F (2020). A review on current anode materials for rechargeable Mg batteries. J. Magnes. Alloys.

[89] Davidson R, Verma A, Santos D, Hao F, Fincher C (2019). Formation of magnesium dendrites during electrodeposition. ACS Energy Lett..

[90] Aurbach D, Gofer Y, Schechter A, Chusid O, Gizbar H (2001). A comparison between the electrochemical behavior of reversible magnesium and lithium electrodes. J. Power Sources.

[91] Muldoon J, Bucur CB, Oliver AG, Sugimoto T, Matsui M (2012). Electrolyte roadblocks to a magnesium rechargeable battery. Energy Environ. Sci..

[92] Deivanayagam R, Ingram BJ, Shahbazian-Yassar R (2019). Progress in development of electrolytes for magnesium batteries. Energy Storage Mater..

[93] Liang Z, Ban C (2021). Strategies to enable reversible magnesium electrochemistry: from electrolytes to artificial solid–electrolyte interphases. Angew. Chem. Int. Ed..

[94] Nam KW, Kim S, Lee S, Salama M, Shterenberg I (2015). The high performance of crystal water containing manganese birnessite cathodes for magnesium batteries. Nano Lett..

[95] Aurbach D, Lu Z, Schechter A, Gofer Y, Gizbar H (2000). Prototype systems for rechargeable magnesium batteries. Nature.

[96] Huie MM, Bock DC, Takeuchi ES, Marschilok AC, Takeuchi KJ (2015). Cathode materials for magnesium and magnesium-ion based batteries. Coord. Chem. Rev..

[97] Mao M, Gao T, Hou S, Wang C (2018). A critical review of cathodes for rechargeable Mg batteries. Chem. Soc. Rev..

[98] Ma Z, MacFarlane DR, Kar M (2019). Mg cathode materials and electrolytes for rechargeable Mg batteries: a review. Batteries Supercaps.

[99] Li Z, Han L, Wang Y, Li X, Lu J (2019). Microstructure characteristics of cathode materials for rechargeable magnesium batteries. Small.

[100] Rashad M, Asif M, Ahmed I, He Z, Yin L (2020). Quest for carbon and vanadium oxide based rechargeable magnesium-ion batteries. J. Magnes. Alloys.

[101] Ling C, Zhang R (2017). Manganese dioxide As rechargeable magnesium battery cathode. Front. Energy Res..

[102] Lee SH, DiLeo RA, Marschilok AC, Takeuchi KJ, Takeuchi ES (2014). Sol gel based synthesis and electrochemistry of magnesium vanadium oxide: a promising cathode material for secondary magnesium ion batteries. ECS Electrochem. Lett..

[103] Zhang T, Tang Y, Guo S, Cao X, Pan A (2020). Fundamentals and perspectives in developing zinc-ion battery electrolytes: a comprehensive review. Energy Environ. Sci..

[104] Wang T, Li C, Xie X, Lu B, He Z (2020). Anode materials for aqueous zinc ion batteries: mechanisms, properties, and perspectives. ACS Nano.

[105] Song M, Tan H, Chao D, Fan HJ (2018). Recent advances in Zn-ion batteries. Adv. Funct. Mater.

[106] Selvakumaran D, Pan A, Liang S, Cao G (2019). A review on recent developments and challenges of cathode materials for rechargeable aqueous Zn-ion batteries. J. Mater. Chem. A.

[107] Zhang N, Chen X, Yu M, Niu Z, Cheng F (2020). Materials chemistry for rechargeable zinc-ion batteries. Chem. Soc. Rev..

[108] Mateos M, Makivic N, Kim YS, Limoges B, Balland V (2020). Accessing the two-electron charge storage capacity of MnO_2_ in mild aqueous electrolytes. Adv. Energy Mater..

[109] Yang J, Cao J, Peng Y, Yang W, Barg S (2020). Unravelling the mechanism of rechargeable aqueous Zn–MnO_2_ batteries: implementation of charging process by electrodeposition of MnO_2_. Chemsuschem.

[110] Wu D, Housel LM, Kim SJ, Sadique N, Quilty CD (2020). Quantitative temporally and spatially resolved X-ray fluorescence microprobe characterization of the manganese dissolution-deposition mechanism in aqueous Zn/α-MnO_2_ batteries. Energy Environ. Sci..

[111] Wu B, Zhang G, Yan M, Xiong T, He P (2018). Graphene scroll-coated α-MnO_2_ nanowires as high-performance cathode materials for aqueous Zn-ion battery. Small.

[112] Pan H, Shao Y, Yan P, Cheng Y, Han KS (2016). Reversible aqueous zinc/manganese oxide energy storage from conversion reactions. Nat. Energy.

[113] Zhang L, Chen L, Zhou X, Liu Z (2015). Morphology-dependent electrochemical performance of zinc hexacyanoferrate cathode for zinc-ion battery. Sci. Rep..

[114] Komaba S, Hasegawa T, Dahbi M, Kubota K (2015). Potassium intercalation into graphite to realize high-voltage/high-power potassium-ion batteries and potassium-ion capacitors. Electrochem. Commun..

[115] Min X, Xiao J, Fang M, Wang W, Zhao Y (2021). Potassium-ion batteries: outlook on present and future technologies. Energy Environ. Sci..

[116] Jian Z, Luo W, Ji X (2015). Carbon electrodes for K-ion batteries. J. Am. Chem. Soc..

[117] Sha M, Liu L, Zhao H, Lei Y (2020). Anode materials for potassium-ion batteries: current status and prospects. Carbon Energy.

[118] Ma L, Lv Y, Wu J, Xia C, Kang Q (2021). Recent advances in anode materials for potassium-ion batteries: a review. Nano Res..

[119] Li W, Bi Z, Zhang W, Wang J, Rajagopalan R (2021). Advanced cathodes for potassium-ion batteries with layered transition metal oxides: a review. J. Mater. Chem. A.

[120] Wu Z, Zou J, Chen S, Niu X, Liu J (2021). Potassium-ion battery cathodes: past, present, and prospects. J. Power Sources.

[121] Dompablo MEA, Ponrouch A, Johansson P, Palacín MR (2020). Achievements, challenges, and prospects of calcium batteries. Chem. Rev..

[122] Gummow RJ, Vamvounis G, Kannan MB, He Y (2018). Calcium-ion batteries: current state-of-the-art and future perspectives. Adv. Mater..

[123] Wang M, Tang Y (2018). A review on the features and progress of dual-ion batteries. Adv. Energy Mater..

[124] Sui Y, Liu C, Masse RC, Neale ZG, Atif M (2020). Dual-ion batteries: the emerging alternative rechargeable batteries. Energy Storage Mater..

[125] Zhang L, Wang H, Zhang X, Tang Y (2021). A review of emerging dual-ion batteries: fundamentals and recent advances. Adv. Funct. Mater..

[126] Obrezkov FA, Shestakov AF, Traven VF, Stevenson KJ, Troshin PA (2019). An ultrafast charging polyphenylamine-based cathode material for high rate lithium, sodium and potassium batteries. J. Mater. Chem. A.

[127] Dong S, Li Z, Rodríguez-Pérez IA, Jiang H, Lu J (2017). A novel coronene//Na_2_Ti_3_O_7_ dual-ion battery. Nano Energy.

[128] Zhang M, Garcia-Araez N, Hector AL (2018). Understanding and development of olivine LiCoPO_4_ cathode materials for lithium-ion batteries. J. Mater. Chem. A.

[129] Kraytsberg A, Ein-Eli Y (2012). Higher, stronger, better… a review of 5 volt cathode materials for advanced lithium-ion batteries. Adv. Energy Mater..

[130] Liang G, Peterson VK, See KW, Guo Z, Pang WK (2020). Developing high-voltage spinel LiNi_0.5_Mn_1.5_O_4_ cathodes for high-energy-density lithium-ion batteries: current achievements and future prospects. J. Mater. Chem. A.

[131] Rozier P, Tarascon JM (2015). Review—Li-rich layered oxide cathodes for next-generation Li-ion batteries: chances and challenges. J. Electrochem. Soc..

[132] Bhatt MD, Lee JY (2019). High capacity conversion anodes in Li-ion batteries: a review. Int. J. Hydrog..

[133] Fang S, Bresser D, Passerini S (2020). Transition metal oxide anodes for electrochemical energy storage in lithium- and sodium-ion batteries. Adv. Energy Mater..

[134] Yang X, Liang HJ, Yu HY, Wang MY, Zhao XX (2020). Recent progresses and challenges of metal sulfides as advanced anode materials in rechargeable sodium-ion batteries. J. Phys. Mater..

[135] Lu Y, Yu L, Lou XW (2018). Nanostructured conversion-type anode materials for advanced lithium-ion batteries. Chem.

[136] Luo P, Zheng C, He J, Tu X, Sun W (2022). Structural engineering in graphite-based metal-ion batteries. Adv. Funct. Mater..

[137] Wang F, Yi J, Wang Y, Wang C, Wang J (2014). Graphite intercalation compounds (GICs): a new type of promising anode material for lithium-ion batteries. Adv. Energy Mater..

[138] Wang M, Zhang F, Lee CS, Tang Y (2017). Low-cost metallic anode materials for high performance rechargeable batteries. Adv. Energy Mater..

[139] Chen X, Li H, Yan Z, Cheng F, Chen J (2019). Structure design and mechanism analysis of silicon anode for lithium-ion batteries. Sci. China Mater..

[140] Zuo X, Zhu J, Müller-Buschbaum P, Cheng YJ (2017). Silicon based lithium-ion battery anodes: a chronicle perspective review. Nano Energy.

[141] Feng K, Li M, Liu W, Kashkooli AG, Xiao X (2018). Silicon-based anodes for lithium-ion batteries: from fundamentals to practical applications. Small.

[142] Yang J, Winter M, Besenhard JO (1996). Small particle size multiphase Li-alloy anodes for lithium-ionbatteries. Solid State Ion..

[143] Huggins RA, Nix WD (2000). Decrepitation model for capacity loss during cycling of alloys in rechargeable electrochemical systems. Ionics.

[144] Chae S, Choi SH, Kim N, Sung J, Cho J (2020). Integration of graphite and silicon anodes for the commercialization of high-energy lithium-ion batteries. Angew. Chem. Int. Ed..

[145] Wilson AM, Way BM, Dahn JR, Buuren T (1995). Nanodispersed silicon in pregraphitic carbons. J. Appl. Phys..

[146] Wang CS, Wu GT, Zhang XB, Qi ZF, Li WZ (1998). Lithium insertion in carbon-silicon composite materials produced by mechanical milling. J. Electrochem. Soc..

[147] Hwa Y, Kim WS, Hong SH, Sohn HJ (2012). High capacity and rate capability of core–shell structured nano-Si/C anode for Li-ion batteries. Electrochim. Acta.

[148] Wen Y, Zhu Y, Langrock A, Manivannan A, Ehrman SH (2013). Graphene-bonded and -encapsulated Si nanoparticles for lithium ion battery anodes. Small.

[149] Zhang C, Kang TH, Yu JS (2018). Three-dimensional spongy nanographene-functionalized silicon anodes for lithium ion batteries with superior cycling stability. Nano Res..

[150] Chan CK, Peng H, Liu G, McIlwrath K, Zhang XF (2008). High-performance lithium battery anodes using silicon nanowires. Nat. Nanotechnol..

[151] Li P, Zhao G, Zheng X, Xu X, Yao C (2018). Recent progress on silicon-based anode materials for practical lithium-ion battery applications. Energy Storage Mater..

[152] Peng K, Jie J, Zhang W, Lee ST (2008). Silicon nanowires for rechargeable lithium-ion battery anodes. Appl. Phys. Lett..

[153] Zhao G, Meng Y, Zhang N, Sun K (2012). Electrodeposited Si film with excellent stability and high rate performance for lithium-ion battery anodes. Mater. Lett..

[154] Yao Y, McDowell MT, Ryu I, Wu H, Liu N (2011). Interconnected silicon hollow nanospheres for lithium-ion battery anodes with long cycle life. Nano Lett..

[155] Steinbach I (2013). Why solidification? Why phase-field?. JOM.

[156] Zheng G, Wang C, Pei A, Lopez J, Shi F (2016). High-performance lithium metal negative electrode with a soft and flowable polymer coating. ACS Energy Lett..

[157] Chen H, Xu H, Zheng B, Wang S, Huang T (2017). Oxide film efficiently suppresses dendrite growth in aluminum-ion battery. ACS Appl. Mater. Interfaces.

[158] Yang Q, Li Q, Liu Z, Wang D, Guo Y (2020). Dendrites in Zn-based batteries. Adv. Mater..

[159] Zheng J, Tang T, Zhao Q, Liu X, Deng Y (2019). Physical orphaning versus chemical instability: is dendritic electrodeposition of Li fatal?. ACS Energy Lett..

[160] Barton JL, Bockris JO (1962). The electrolytic growth of dendrites from ionic solutions. Proc. R. Soc. Lond. A.

[161] He Y, Ren X, Xu Y, Engelhard MH, Li X (2019). Origin of lithium whisker formation and growth under stress. Nat. Nanotechnol..

[162] Wang R, Cui W, Chu F, Wu F (2020). Lithium metal anodes: present and future. J. Energy Chem..

[163] Hundekar P, Jain R, Lakhnot AS, Koratkar N (2020). Recent advances in the mitigation of dendrites in lithium-metal batteries. J. Appl. Phys..

[164] Cao D, Sun X, Li Q, Natan A, Xiang P (2020). Lithium dendrite in all-solid-state batteries: growth mechanisms, suppression strategies, and characterizations. Matter.

[165] Wu F, Yushin G (2017). Conversion cathodes for rechargeable lithium and lithium-ion batteries. Energy Environ. Sci..

[166] Badway F, Cosandey F, Pereira N, Amatucci GG (2003). Carbon metal fluoride nanocomposites: high-capacity reversible metal fluoride conversion materials as rechargeable positive electrodes for Li batteries. J. Electrochem. Soc..

[167] Badway F, Mansour AN, Pereira N, Al-Sharab JF, Cosandey F (2007). Structure and electrochemistry of copper fluoride nanocomposites utilizing mixed conducting matrices. Chem. Mater..

[168] Omenya F, Zagarella NJ, Rana J, Zhang H, Siu C (2019). Intrinsic challenges to the electrochemical reversibility of the high energy density copper(II) fluoride cathode material. ACS Appl. Energy Mater..

[169] Wang F, Robert R, Chernova NA, Pereira N, Omenya F (2011). Conversion reaction mechanisms in lithium ion batteries: study of the binary metal fluoride electrodes. J. Am. Chem. Soc..

[170] Huang Q, Turcheniuk K, Ren X, Magasinski A, Song AY (2019). Cycle stability of conversion-type iron fluoride lithium battery cathode at elevated temperatures in polymer electrolyte composites. Nat. Mater..

[171] Conte DE, Pinna N (2014). A review on the application of iron(III) fluorides as positive electrodes for secondary cells. Mater. Renew. Sustain. Energy.

[172] Fan X, Hu E, Ji X, Zhu Y, Han F (2018). High energy-density and reversibility of iron fluoride cathode enabled via an intercalation-extrusion reaction. Nat. Commun..

[173] Barghamadi M, Kapoor A, Wen C (2013). A review on Li-S batteries as a high efficiency rechargeable lithium battery. J. Electrochem. Soc..

[174] Li T, Bai X, Gulzar U, Bai YJ, Capiglia C (2019). A comprehensive understanding of lithium–sulfur battery technology. Adv. Funct. Mater..

[175] Dörfler S, Walus S, Locke J, Fotouhi A, Auger DJ (2021). Recent progress and emerging application areas for lithium–sulfur battery technology. Energy Technol..

[176] Li G, Wang S, Zhang Y, Li M, Chen Z (2018). Revisiting the role of polysulfides in lithium–sulfur batteries. Adv. Mater..

[177] Zheng D, Wang G, Liu D, Si J, Ding T (2018). The progress of Li–S batteries—understanding of the sulfur redox mechanism: dissolved polysulfide ions in the electrolytes. Adv. Mater. Technol..

[178] Dahn JR, Burns JC, Stevens DA (2016). Importance of coulombic efficiency measurements in R&D efforts to obtain long-lived Li-ion batteries. Electrochem. Soc. Interface.

[179] Xin S, Gu L, Zhao NH, Yin YX, Zhou LJ (2012). Smaller sulfur molecules promise better lithium–sulfur batteries. J. Am. Chem. Soc..

[180] Li G, Sun J, Hou W, Jiang S, Huang Y (2016). Three-dimensional porous carbon composites containing high sulfur nanoparticle content for high-performance lithium–sulfur batteries. Nat. Commun..

[181] Yang J, Yang X, Cheong JL, Zaghib K, Trudeau ML (2021). Nanoboxes with a porous MnO core and amorphous TiO_2_ shell as a mediator for lithium–sulfur batteries. J. Mater. Chem. A.

[182] Wang P, Xi B, Huang M, Chen W, Feng J (2021). Emerging catalysts to promote kinetics of lithium–sulfur batteries. Adv. Energy Mater..

[183] Liu D, Zhang C, Zhou G, Lv W, Ling G (2018). Catalytic effects in lithium–sulfur batteries: promoted sulfur transformation and reduced shuttle effect. Adv. Sci..

[184] Zhou T, Lv W, Li J, Zhou G, Zhao Y (2017). Twinborn TiO_2_–TiN heterostructures enabling smooth trapping–diffusion–conversion of polysulfides towards ultralong life lithium–sulfur batteries. Energy Environ. Sci..

[185] Gupta A, Sivaram S (2019). Separator membranes for lithium–sulfur batteries: design principles, structure, and performance. Energy Technol..

[186] Rana M, Li M, Huang X, Luo B, Gentle I (2019). Recent advances in separators to mitigate technical challenges associated with re-chargeable lithium sulfur batteries. J. Mater. Chem. A.

[187] Zhao Q, Hao Z, Tang J, Xu X, Liu J (2021). Cation-selective separators for addressing the lithium–sulfur battery challenges. Chemsuschem.

[188] Zhao M, Li BQ, Peng HJ, Yuan H, Wei JY (2020). Lithium–sulfur batteries under lean electrolyte conditions: challenges and opportunities. Angew. Chem. Int. Ed..

[189] Imanishi N, Yamamoto O (2019). Perspectives and challenges of rechargeable lithium–air batteries. Mater. Today Adv..

[190] Manthiram A, Li L (2015). Hybrid and aqueous lithium-air batteries. Adv. Energy Mater..

[191] Li CS, Sun Y, Gebert F, Chou SL (2017). Current progress on rechargeable magnesium–air battery. Adv. Energy Mater..

[192] Mori R (2020). Recent developments for aluminum–air batteries. Electrochem. Energ. Rev..

[193] Gu P, Zheng M, Zhao Q, Xiao X, Xue H (2017). Rechargeable zinc–air batteries: a promising way to green energy. J. Mater. Chem. A.

[194] Kulkarni A, Siahrostami S, Patel A, Nørskov JK (2018). Understanding catalytic activity trends in the oxygen reduction reaction. Chem. Rev..

[195] Wang X, Li Z, Qu Y, Yuan T, Wang W (2019). Review of metal catalysts for oxygen reduction reaction: from nanoscale engineering to atomic design. Chem.

[196] Tian X, Lu XF, Xia BY, Lou XW (2020). Advanced electrocatalysts for the oxygen reduction reaction in energy conversion technologies. Joule.

[197] Mitchell S, Pérez-Ramírez J (2020). Single atom catalysis: a decade of stunning progress and the promise for a bright future. Nat. Commun..

[198] Han J, Bian J, Sun C (2020). Recent advances in single-atom electrocatalysts for oxygen reduction reaction. Research.

[199] Suen NT, Hung SF, Quan Q, Zhang N, Xu YJ (2017). Electrocatalysis for the oxygen evolution reaction: recent development and future perspectives. Chem. Soc. Rev..

[200] Lee WH, Ko YJ, Kim JY, Min BK, Hwang YJ (2020). Single-atom catalysts for the oxygen evolution reaction: recent developments and future perspectives. Chem. Commun..

[201] Tomboc GM, Yu P, Kwon T, Lee K, Li J (2020). Ideal design of air electrode—a step closer toward robust rechargeable Zn–air battery. APL Mater..

[202] Wang YJ, Fang B, Zhang D, Li A, Wilkinson DP (2018). A review of carbon-composited materials as air-electrode bifunctional electrocatalysts for metal–air batteries. Electrochem. Energ. Rev..

[203] Ren S, Duan X, Liang S, Zhang M, Zheng H (2020). Bifunctional electrocatalysts for Zn–air batteries: recent developments and future perspectives. J. Mater. Chem. A.

[204] Park GS, Lee JS, Kim ST, Park S, Cho J (2013). Porous nitrogen doped carbon fiber with churros morphology derived from electrospun bicomponent polymer as highly efficient electrocatalyst for Zn–air batteries. J. Power Sources.

[205] Wang J, Wu H, Gao D, Miao S, Wang G (2015). High-density iron nanoparticles encapsulated within nitrogen-doped carbon nanoshell as efficient oxygen electrocatalyst for zinc–air battery. Nano Energy.

[206] Neburchilov V, Wang H, Martin JJ, Qu W (2010). A review on air cathodes for zinc–air fuel cells. J. Power Sources.

[207] Jung JW, Cho SH, Nam JS, Kim ID (2020). Current and future cathode materials for non-aqueous Li-air (O_2_) battery technology—a focused review. Energy Storage Mater..

[208] Yin XP, Wang HJ, Tang SF, Lu XL, Shu M (2018). Engineering the coordination environment of single-atom platinum anchored on graphdiyne for optimizing electrocatalytic hydrogen evolution. Angew. Chem. Int. Ed..

[209] Guo X, Hu X, Wu D, Jing C, Liu W (2019). Tuning the bifunctional oxygen electrocatalytic properties of core–shell Co_3_O_4_@NiFe LDH catalysts for Zn–air batteries: effects of interfacial cation valences. ACS Appl. Mater. Interfaces.

[210] Energizer zinc air prismatic handbook. (Energizer Battery Manufacturing). https://data.energizer.com/pdfs/zincairprismatichandbook.pdf. Accessed 25 July 2021

[211] Bankole OE, Gong C, Lei L (2013). Battery recycling technologies: recycling waste lithium ion batteries with the impact on the environment in-view. J. Ecol. Environ..

[212] Lee JS, Kim ST, Cao R, Choi NS, Liu M (2011). Metal–air batteries with high energy density: Li–air versus Zn–air. Adv. Energy Mater..

[213] Deng K, Zeng Q, Wang D, Liu Z, Wang G (2020). Nonflammable organic electrolytes for high-safety lithium-ion batteries. Energy Storage Mater..

[214] Gond R, Ekeren W, Mogensen R, Naylor AJ, Younesi R (2021). Non-flammable liquid electrolytes for safe batteries. Mater. Horiz..

[215] Chen J, Naveed A, Nuli Y, Yang J, Wang J (2020). Designing an intrinsically safe organic electrolyte for rechargeable batteries. Energy Storage Mater..

[216] Pan Z, Liu X, Yang J, Li X, Liu Z (2021). Aqueous rechargeable multivalent metal-ion batteries: advances and challenges. Adv. Energy Mater..

[217] Li W, Dahn JR, Wainwright DS (1994). Rechargeable lithium batteries with aqueous electrolytes. Science.

[218] Ma L, Chen S, Long C, Li X, Zhao Y (2019). Achieving high-voltage and high-capacity aqueous rechargeable zinc ion battery by incorporating two-species redox reaction. Adv. Energy Mater..

[219] Chun SE, Evanko B, Wang X, Vonlanthen D, Ji X (2015). Design of aqueous redox-enhanced electrochemical capacitors with high specific energies and slow self-discharge. Nat. Commun..

[220] Zhang H, Liu X, Li H, Hasa I, Passerini S (2021). Challenges and strategies for high-energy aqueous electrolyte rechargeable batteries. Angew. Chem. Int. Ed..

[221] Wang Y, Meng X, Sun J, Liu Y, Hou L (2020). Recent progress in “water-in-salt” electrolytes toward non-lithium based rechargeable batteries. Front. Chem..

[222] Chao D, Zhou W, Xie F, Ye C, Li H (2020). Roadmap for advanced aqueous batteries: from design of materials to applications. Sci. Adv..

[223] Yang C, Chen J, Qing T, Fan X, Sun W (2017). 4.0 V Aqueous Li-ion batteries. Joule.

[224] Zhao Q, Stalin S, Zhao CZ, Archer LA (2020). Designing solid-state electrolytes for safe, energy-dense batteries. Nat. Rev. Mater..

[225] Hansen EJ, Liu J (2021). Materials and structure design for solid-state zinc-ion batteries: a mini-review. Front. Energy Res..

[226] Li Z, Liu P, Zhu K, Zhang Z, Si Y (2021). Solid-state electrolytes for sodium metal batteries. Energy Fuels.

[227] Bachman JC, Muy S, Grimaud A, Chang HH, Pour N (2016). Inorganic solid-state electrolytes for lithium batteries: mechanisms and properties governing ion conduction. Chem. Rev..

[228] Meesala Y, Jena A, Chang H, Liu RS (2017). Recent advancements in Li-ion conductors for all-solid-state Li-ion batteries. ACS Energy Lett..

[229] Zheng F, Kotobuki M, Song S, Lai MO, Lu L (2018). Review on solid electrolytes for all-solid-state lithium-ion batteries. J. Power Sources.

[230] Manthiram A, Yu X, Wang S (2017). Lithium battery chemistries enabled by solid-state electrolytes. Nat. Rev. Mater..

[231] Grady ZA, Wilkinson CJ, Randall CA, Mauro JC (2020). Emerging role of non-crystalline electrolytes in solid-state battery research. Front. Energy Res..

[232] Famprikis T, Canepa P, Dawson JA, Islam MS, Masquelier C (2019). Fundamentals of inorganic solid-state electrolytes for batteries. Nat. Mater..

[233] Richards WD, Miara LJ, Wang Y, Kim JC, Ceder G (2016). Interface stability in solid-state batteries. Chem. Mater..

[234] Banerjee A, Wang X, Fang C, Wu EA, Meng YS (2020). Interfaces and interphases in all-solid-state batteries with inorganic solid electrolytes. Chem. Rev..

[235] Zhou W, Wang S, Li Y, Xin S, Manthiram A (2016). Plating a dendrite-free lithium anode with a polymer/ceramic/polymer sandwich electrolyte. J. Am. Chem. Soc..

[236] Du F, Zhao N, Li Y, Chen C, Liu Z (2015). All solid state lithium batteries based on lamellar garnet-type ceramic electrolytes. J. Power Sources.

[237] Zhang Y, Chen F, Yang D, Zha W, Li J (2017). High capacity all-solid-state lithium battery using cathodes with three-dimensional Li^+^ conductive network. J. Electrochem. Soc..

[238] Gutiérrez-Pardo A, Martinez AIP, Otaegui L, Schneider M, Roters A (2018). Will the competitive future of solid state Li metal batteries rely on a ceramic or a composite electrolyte?. Sustain. Energy Fuels.

[239] Song SW, Lee KC, Park HY (2016). High-performance flexible all-solid-state microbatteries based on solid electrolyte of lithium boron oxynitride. J. Power Sources.

[240] Balaish M, Gonzalez-Rosillo JC, Kim KJ, Zhu Y, Hood ZD (2021). Processing thin but robust electrolytes for solid-state batteries. Nat. Energy.

[241] Yue L, Ma J, Zhang J, Zhao J, Dong S (2016). All solid-state polymer electrolytes for high-performance lithium ion batteries. Energy Storage Mater..

[242] Arya A, Sharma AL (2020). A glimpse on all-solid-state Li-ion battery (ASSLIB) performance based on novel solid polymer electrolytes: a topical review. J. Mater. Sci..

[243] Jeong K, Park S, Lee SY (2019). Revisiting polymeric single lithium-ion conductors as an organic route for all-solid-state lithium ion and metal batteries. J. Mater. Chem. A.

[244] Yi C, Liu W, Li L, Dong H, Liu J (2019). Polymer-in-salt solid electrolytes for lithium-ion batteries. Funct. Mater. Lett..

[245] Yao P, Yu H, Ding Z, Liu Y, Lu J (2019). Review on polymer-based composite electrolytes for lithium batteries. Front. Chem..

[246] Zheng Y, Yao Y, Ou J, Li M, Luo D (2020). A review of composite solid-state electrolytes for lithium batteries: fundamentals, key materials and advanced structures. Chem. Soc. Rev..

[247] Preliminary cell data sheet, 3D silicon^TM^ lithium-ion rechargeable cell model EX1-395578A (Enovix, 2021). https://www.enovix.com/#products. Accessed 25 July 2021

[248] Ultra light lithium sulfur pouch cell (Oxis Energy, 2019). https://oxisenergy.com/products. Accessed 25 July 2021

[249] Achievements - research & development—HiNa battery technology Co. Ltd. (HiNa Battery, 2021). https://www.hinabattery.com/en/index.php?catid=15. Accessed 25 July 2021

[250] Strong performance (Faradion, 2021). https://www.faradion.co.uk/technology-benefits/strong-performance. Accessed 29 July 2021

[251] Fiche technique Pack LMP® 63 (Blue Solutions by Bolloré, 2019). https://blue-storage.com/en/our-files. Accessed 25 July 2021

[252] Krause FC, Ruiz JP, Jones SC, Brandon EJ, Darcy EC (2021). Performance of commercial Li-ion cells for future NASA missions and aerospace applications. J. Electrochem. Soc..

[253] Powin stacks datasheet (Powin Energy, 2020). https://powin.com/products/stacks. Accessed 29 July 2021

[254] Tesla powerwall limited warranty (Tesla, 2021). https://www.tesla.com/support/energy/powerwall/documents. Accessed 29 July 2021

[255] Tesla powerwall technical specifications (Tesla, 2021). https://www.tesla.com/support/energy/powerwall/documents. Accessed 29 July 2021

[256] eZ8 (Cegasa, 2018). https://www.cegasa.com/en/ez8. Accessed 25 July 2021

[257] Rudola A, Rennie AJR, Heap R, Meysami SS, Lowbridge A (2021). Commercialisation of high energy density sodium-ion batteries: Faradion’s journey and outlook. J. Mater. Chem. A.

[258] BatPaC: battery manufacturing cost estimation (Argonne National Laboratory, 2021). https://www.anl.gov/partnerships/batpac-battery-manufacturing-cost-estimation. Accessed 25 July 2021

[259] Peters JF, Cruz AP, Weil M (2019). Exploring the economic potential of sodium-ion batteries. Batteries.

[260] Smekens J, Gopalakrishnan R, Steen NV, Omar N, Hegazy O (2016). Influence of electrode density on the performance of Li-ion batteries: experimental and simulation results. Energies.

[261] Lv W, Wang Z, Cao H, Sun Y, Zhang Y (2018). A critical review and analysis on the recycling of spent lithium-ion batteries. ACS Sustain. Chem. Eng..

[262] Zhao X, Yang H, Wang Y, Sha Z (2019). Review on the electrochemical extraction of lithium from seawater/brine. J. Electroanal. Chem..

[263] Li Z, Li C, Liu X, Cao L, Li P (2021). Continuous electrical pumping membrane process for seawater lithium mining. Energy Environ. Sci..

[264] Cerdas F, Titscher P, Bognar N, Schmuch R, Winter M (2018). Exploring the effect of increased energy density on the environmental impacts of traction batteries: a comparison of energy optimized lithium-ion and lithium-sulfur batteries for mobility applications. Energies.

[265] Wang Z, Ning X, Zhu K, Hu J, Yang H (2019). Evaluating the thermal failure risk of large-format lithium-ion batteries using a cone calorimeter. J. Fire Sci..

[266] A new breed of battery - investor presentation (Solid Power, 2021). https://investors.solidpowerbattery.com/home/default.aspx. Accessed 25 July 2021

[267] Toshiba develops world’s first aqueous lithium-ion battery with nonflammable electrolyte (Toshiba, 2020). https://www.global.toshiba/ww/technology/corporate/rdc/rd/topics/20/2011-01.html. Accessed 25 July 2021

[268] Zinc8 energy solutions (Zinc8 Energy Solutions, 2021). https://www.zinc8energy.com. Accessed 29 July 2021

[269] Form energy (Form Energy, 2021). https://formenergy.com. Accessed 29 July 2021

[270] Saft lithium batteries Selector guide (Saft Batteries, 2020). https://www.saftbatteries.com/products-solutions/products/ls-lsh-lsp. Accessed 29 July 2021

[271] Métalectrique (Métalectrique, 2021). https://www.metalectrique.com. Accessed 25 July 2021

[272] MGV (MGV, 2021). https://www.mgv.jp. Accessed 29 July 2021

[273] MgBOX magnesium air battery (Eco Marine Power, 2021). https://www.ecomarinepower.com/en/mgbox-air-battery. Accessed 29 July 2021

[274] Emergency magnesium air cell (Fujikura Composites, 2019). https://www.fujikuracomposites.jp/en/focus/research/wattsatt/. Accessed 29 July 2021

[275] Quinn JB, Waldmann T, Richter K, Kasper M, Wohlfahrt-Mehrens M (2018). Energy density of cylindrical Li-ion cells: a comparison of commercial 18650 to the 21700 cells. J. Electrochem. Soc..

[276] Waldmann T, Scurtu RG, Richter K, Wohlfahrt-Mehrens M (2020). 18650 vs. 21700 Li-ion cells—a direct comparison of electrochemical, thermal, and geometrical properties. J. Power Sources.

